# Neuroinflammatory and Motor Alterations in LRRK2*G2019S Transgenic Mice Without Enhanced Vulnerability to Aging or Chronic MPTP‐Induced Nigrostriatal Neurodegeneration

**DOI:** 10.1111/jnc.70547

**Published:** 2026-09-09

**Authors:** Roberto García‐Swinburn, Laura Morón‐Márquez, Carmen Conde‐Naranjo, Ernesto García‐Roldán, Isabel Espadas, Alfonso Rodríguez‐Gil, Reposo Ramírez‐Lorca, Javier Villadiego, Juan José Toledo‐Aral

**Affiliations:** ^1^ Instituto de Biomedicina de Sevilla (IBiS) Hospital Universitario Virgen del Rocío/CSIC/Universidad de Sevilla Sevilla Spain; ^2^ Departamento de Fisiología Médica y Biofísica, Facultad de Medicina Universidad de Sevilla Sevilla Spain; ^3^ Servicio de Neurología y Neurofisiología Clínica Hospital Universitario Virgen del Rocío Sevilla Spain; ^4^ Centro de Investigación Biomédica en Red sobre Enfermedades Neurodegenerativas (CIBERNED) Madrid Spain

**Keywords:** aging, LRRK2, MPTP, neurodegeneration, Parkinson's disease

## Abstract

LRRK2 G2019S mutation is the most common genetic cause of Parkinson's disease (PD) producing clinical manifestations similar to sporadic PD patients, hinting at the relevance of this mutation in the pathophysiological mechanisms of the disease. However, its role potentiating nigrostriatal neurodegeneration, particularly in response to mitochondrial dysfunction, remains unclear. Here, using histological, neurochemical and behavioral analyzes, we provide a complete characterization of a mouse model expressing the human LRRK2 G2019S mutation (hLRRK2*G2019S mice), in the context of aging and toxin‐induced parkinsonism. hLRRK2*G2019S young adult mice showed significant microglial activation and deficits in motor coordination, whereas aged transgenic displayed a hyperactive phenotype, despite the absence of evidence for nigrostriatal neurodegeneration in either young adult or aged animals. Furthermore, we assessed the vulnerability of LRRK2*G2019S transgenic mice to the chronic exposure to the mitochondrial complex I inhibitor 1‐methyl‐4‐phenyl‐1,2,3,6‐tetrahydropyridine (MPTP). Notably, no exacerbation of nigrostriatal neurodegeneration was observed in hLRRK2*G2019S mice, suggesting that this mutation does not increase sensitivity to mitochondrial stress. However, hLRRK2*G2019S MPTP‐treated mice present alterations, relative to WT counterparts, in the glial response induced by the neurotoxic treatment. These findings suggest that the G2019S mutation modulates the neuroimmune response but does not exacerbate nigrostriatal neurodegeneration in response to mitochondrial dysfunction. This highlights the mutation's complex role in PD pathophysiology.

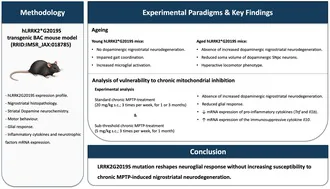

## Graphical abstract

LRRK2 G2019S mutation is the most common genetic cause of Parkinson's disease but its contribution to nigrostriatal neurodegeneration, particularly in response to mitochondrial dysfunction, remains unclear. hLRRK2*G2019S transgenic mice exhibited early microglial activation and motor alterations, but not increased dopaminergic nigrostriatal neurodegeneration, either in young or aged mice. Furthermore, hLRRK2G2019S mice did not exhibit increased susceptibility of SNpc dopaminergic neurons to long‐term mitochondrial inhibition induced by chronic MPTP treatment, but instead displayed a reduced neuroglial response associated with dopaminergic damage. These findings indicate that the LRRK2 G2019S mutation reshapes neuroglial responses without increasing susceptibility to chronic MPTP‐induced nigrostriatal neurodegeneration.

## 1 Introduction

Parkinson's disease (PD) is characterized by the degeneration of dopaminergic substantia nigra pars compacta (SNpc) neurons that results in striatal denervation and consequently the typical motor symptoms. Mutations in leucine‐rich repeat kinase 2 [LRRK2; PARK8 locus encoding dardarin protein; (Funayama et al. 2002; Paisán‐Ruíz et al. 2004)] are the most common genetic cause of PD. LRRK2 mutations present autosomal dominant inheritance pattern (Correia Guedes et al. 2010; Mata et al. 2023), indicating a possible negative gain of function of the mutated protein. Furthermore, common genetic variance at the LRRK2 locus has been identified as a risk factor for PD, indicating that this gene is a major actor in the pathophysiology of this neurodegenerative disease (Ross et al. 2011; Satake et al. 2009; Simón‐Sánchez et al. 2009). Among the different LRRK2 mutations, G2019S is the most prevalent and better studied, affecting the serine/threonine kinase domain of the LRRK2 protein and increasing kinase activity (West et al. 2005). This mutation shows a low, but variable, lifetime penetrance, ranging from 17% to 80%, which differs across ethnic groups and increases with aging (Lee et al. 2017; Kmiecik et al. 2024; Healy et al. 2008). Clinical manifestation of parkinsonism in LRRK2 G2019S carriers are very similar to sporadic PD patients, with motor and non‐motor symptoms and good response to levodopa, suggesting a relevant role of this mutation in the common pathophysiological mechanisms of the disease (Taymans et al. 2023; Seegobin et al. 2020).

LRRK2 mutations, particularly the G2019S variant, have been related to different cellular functions that contribute to the neurodegenerative process, such as vesicle trafficking, autophagy and mitochondrial function (West et al. 2005; Mortiboys et al. 2010; Taymans et al. 2023). Specifically, increased kinase activity produced by LRRK2 mutations has been shown to alter mitochondrial dynamics, promoting mitochondrial fragmentation and reducing bioenergetic efficiency (Valderhaug et al. 2024; Ilieva et al. 2024). Different studies have also explored, using animal models, the effects of LRKK2 mutations on the nigrostriatal pathway (see for review Seegobin et al. 2020). Rodent LRRK2 knock‐out did not show relevant phenotypes that mimic parkinsonian features, without loss of dopaminergic SNpc neurons, significant motor alterations, or increased sensitivity to the mitochondrial inhibitor 1‐methyl‐4‐phenyl‐1,2,3,6‐tetrahydropyridine (MPTP) (Andres‐Mateos et al. 2009; Tong et al. 2010; Hinkle et al. 2012). In addition, different works have addressed the potential toxic gain of function of the LRRK2 G2019S mutation in the nigrostriatal pathway in transgenic or knock‐in rodents. Overall, LRRK2 G2019S expressing animals did not present clear parkinsonian‐like phenotypes, ranging from the complete absence of neurodegeneration in the nigrostriatal pathway (Garcia‐Miralles et al. 2015) to subtle alterations in dopaminergic neurons and minor motor deficits (Ramonet et al. 2011; Yue et al. 2015; Chou et al. 2014; Melrose et al. 2010; Volta et al. 2015).

In this work, we report a comprehensive histological, neurochemical and behavioral characterization of a novel Bacterial Artificial Chromosome (BAC)‐driven LRRK2 G2019S mouse model (hLRRK2*G2019S mice), that express the human PD mutation with its regulatory elements. This transgenic hLRRK2*G2019S mouse model was generated by the Michael J. Fox foundation and is available to the scientific community (Baptista et al. 2013; Li et al. 2024; Khan et al. 2021). Young adult hLRRK2*G2019S mice display significant microglial activation, both in the striatum and SNpc, and deficits in gait coordination. Notably, this occurs without significant evidences of nigrostriatal neurodegeneration, maintaining the number of dopaminergic SNpc neurons, the striatal innervation or dopamine (DA) content respect to their WT counterparts. Furthermore, aging did not exacerbate nigrostriatal neurodegeneration of transgenic mice. To study whether hLRRK2 G2019S mutation would increase the susceptibility of dopaminergic SNpc neurons to mitochondrial inhibition, hLRRK2*G2019S mice were subjected to chronic treatment with the dopaminergic complex I blocker neurotoxin MPTP. Mice expressing hLRRK2 G2019S mutation showed similar levels of MPTP‐induced nigrostriatal neurodegeneration and motor deficits than wild type (WT) mice, indicating that this mutation does not increase susceptibility to dopaminergic degeneration in an experimental model of mitochondrial dysfunction.

## 2 Materials and Methods

### 2.1 Animal Care and MPTP Treatment

Male and females hLRRK2*G2019S mice [C57BL/6J‐Tg(LRRK2*G2019S)2AMjff/J, JAX strain #018785, RRID:IMSR_JAX:018785], young adults (3–6 months old) or aged (16–18 months old), were housed in a regulated temperature environment (22°C ± 1°C) on a 12 h light/dark cycle, with ad libitum access to food and water in both the animal facilities of the IBiS or Animal Experimentation Centre‐Oscar Pintado (CITIUS, University of Seville, Spain). Transgenic animals were produced in C57Bl/6J genetic backgrounds, so, in order to maintain a homogeneous background, mutant animals were crossbred with C57BL/6J mice (JAX strain #000664, RRID:IMSR_JAX:000664) for more than nine generations. hLRRK2*G2019S mouse model was produced by Dr. Kuldip Dave, from Michael J. Fox Foundation (Baptista et al. 2013). To produce this strain, a 188 Kb BAC containing the entire human LRRK2 gene was modified by targeted mutation of the LRRK2 locus to harbor the LRRK2 G2019S mutation. The transgene was inserted in chromosome 2 and mouse genotyping was performed by the General Research Service for Biology (CITIUS, University of Seville, Spain) following the protocols indicated by the Jackson Laboratory (“JAX 018785 ‐ Strain Details”).

hLRRK2*G2019S or WT mice were rendered parkinsonian by chronic subcutaneous (s.c.; scruff) administration of 20 mg/kg MPTP [Sigma‐Aldrich, M0896] three times per week for 1 or 3 months, as previously described (Villadiego, Muñoz‐Manchado, et al. 2023; Muñoz‐Manchado et al. 2016), or by a subthreshold dose of 5 mg/kg, 3 times per week for 1 month. MPTP was dissolved in 0.9% NaCl at a concentration of 8 mg/mL for the 20 mg/kg dose, or 2 mg/mL for the 5 mg/kg subthreshold dose. Injection volume was adjusted according to body weight (2.5 μL/g). Saline controls received 0.9% NaCl (Sigma‐Aldrich, S9625) injections on the same schedule as MPTP‐treated animals (injection volume, 2.5 μL/g). The chronic MPTP, or saline solution, treatment did not produce mortality, weight loss, dehydration, or any sign of distress (assessed by the appearance of piloerection, coat staring, ocular and nasal discharges, or aggressive behavior). All behavioral studies were performed 3–4 days after the last dose of MPTP, and finally, mice were euthanized at the end of phenotyping following the appropriate procedures required for tissue processing. Mice were euthanized under deep anesthesia induced by a combination of 100 mg/kg ketamine (Pfizer, Ketolar 47 034) and 10 mg/kg xylazine (Bayer, Rompun 1977 ESP), for intracardial perfusion, or by cervical dislocation, for fresh tissue extraction. All experiments were performed according to the European Directive 2010/63/EU and the Spanish RD/53/2013 for the protection of animals used for scientific purposes. The study was approved by the Animal Research Committee of the University Hospital Virgen del Rocio and the Animal Experimentation Ethics Committee of University of Seville (ethics committee registered agreement numbers 04/04/2019/062 and 11/07/2024/103).

### 2.2 Behavioral Analysis

Behavioral tests were conducted in the morning (09:00–13:00 h) by the same experimenter and always in the same order. First openfield tests, then 1–3 days later the footprint assay (training included). Block randomization was used to minimize potential biases. To this end, hLRRK2*G2019S mice and WT littermates were housed in the same cages. For saline‐ and MPTP‐treated groups, animals were randomly mixed during behavioral testing. Experimenters were blinded to genotype during testing but not to MPTP treatment for safety reasons. For data analysis, experimenters were blinded to all experimental factors. Each mouse was considered an independent statistical unit and was tested only once.

Openfield tests was performed using Biobserve Viewer software (Biobserve), analyzing live video recordings of mice freely exploring an arena measuring 50 × 50 cm with 45 cm‐high walls for 30 min. Videos were recorded in a dark and quiet environment using infrared cameras. Mice were tracked as dark pixels against a light background. Rearing activity was measured using vertically installed infrared beams positioned 5 cm above the floor, with each beam interruption corresponding to a rearing event (standing on hind limbs). Rearing duration was calculated as the mean duration per rearing event for each mouse. Mice were placed in the arena and allowed to habituate for 10 min before recording began. Between sessions, arenas were cleaned using an odorless detergent solution [2% Derquim LM02 (Panreac‐Aplichem, 502601) in water], followed by 70% ethanol (UN1170, Alcoholes del Sur S.A.), and air‐dried for 5 min. Activity was measured as time spent at any movement speed. Data were represented as bar plots representing means of total track length, average speed, activity, total number of rearings, average rearing duration per event, and time spent at slow velocity (< 20 cm/s), medium‐paced velocity (20–60 cm/s) and high velocity (> 60 cm/s). No animals were excluded due to technical reasons.

Footprint assay was carried out to assess gait coordination as previously described (Barlow et al. 1996; Muñoz‐Manchado et al. 2016). Mice were trained 2–3 days before testing by placing them in a dark corridor (10 × 10 × 40 cm) ending in a closed box containing a peanut butter reward. The test was carried out by painting forelimbs in blue and hindlimbs in red with nontoxic, washable finger paint (JOVI; P560S) and a soft fine painting brush. Mice were then placed in the corridor lined with a clean sheet of white paper. Only steps corresponding to a regular walk at uniform speed (i.e., no sudden stops or running) and along a straight trajectory (i.e., no abrupt changes in direction) were analyzed to minimize variability. Runs in which the animal deviated from a straight path, or produced fewer than three clearly identifiable steps were excluded. The first and last pair of steps were also excluded from the analysis. Stride length was measured manually using a ruler, and the mean stride length was calculated from at least three consecutive steps. Gait coordination was quantified as the percentage of stride length variation for forelimbs and hindlimbs separately. This metric was calculated by subtracting the minimum from the maximum stride length for each limb, averaging left and right values, and dividing the resulting value by the mean stride length. Data were represented as mean stride length and percentage of stride variation with respect to mean.

### 2.3 Tissue Processing

In the case of brains used for peroxidase‐based immunohistochemistry and biochemical analysis, mice were euthanized by cervical dislocation and brains were dissected in two brain hemispheres (at the striatal level) and a whole midbrain with the help of a steel mouse brain matrix (Stoelting, 51 386). One hemisphere and the whole midbrain were fixed in 4% paraformaldehyde (PFA, Sigma‐Aldrich, P6148) in phosphate‐buffered saline (PBS; NaCl 0.137 M, KCl 0.0027M, Na_2_HPO_4_ 0.01 M, KH_2_PO_4_ 0.0018M, pH = 7.4; Sigma‐Aldrich, respectively S9625, P4504, S0876, P5379) overnight (o/n) at 4°C, cryoprotected in 30% sucrose (Sigma‐Aldrich, S8501) in PBS until sinking, included in Optimum Cutting Temperature compound (OCT, Tissue‐Tek, 4583) and stored at −80°C (no longer than 6 months) until their use for sectioning (see below). The other hemistriatum was stored at −80°C (no longer than 3 months) until its use for catecholamine measurements (see point 2.5). Regarding immunofluorescent stainings, mice were anesthetized as described above and perfused intracardially with 50 mL of PBS and 50 mL of 4% PFA in PBS using a high precision peristaltic pump (Ismatec, ISM940D). Brains were immediately removed and post‐fixed in 4% PFA (Sigma) in PBS at 4°C for 2 h. After that, brains were cryoprotected, included in OCT (Tissue‐Tek) and stored as described above. Coronal sections (30 μm thick) were cut on a cryostat (Leica, CM3050S), collected serially (5) and stored at −20°C (no longer than 3 months) in cryoprotective solution [30% glycerol and 30% ethylene glycol (Sigma‐Aldrich, respectively G7757, 293 237) in PBS] until their use for histological staining (see point 2.4). For RNA extraction, mice were euthanized by cervical dislocation, and the brains were rapidly removed. The striatum and ventral mesencephalon were immediately dissected using an ice‐cold stainless‐steel brain matrix (Stoelting, 51 386) and a stereoscopic microscope (Olympus SZX16). Dissected tissues were placed in 300 μL of TRIzol Reagent (Thermo Fisher Scientific, 15596026) and processed for RNA extraction as detailed below.

### 2.4 Histological Staining

Free‐floating immunofluorescence was performed using 30 μm brain slices and gentle agitation on an orbital shaker (FINE‐PCR SH‐30, Fine Mold Precision Inc.). Unless otherwise stated, all steps were carried out at room temperature. Incubations with fluorescent secondary antibodies were performed under dim light to minimize photobleaching. Sections were washed twice for 5 min in PBS and permeabilized in PBTx 0.3 (0.3% Triton X‐100 [Sigma‐Aldrich, T8785] in PBS, pH 7.4) for 15 min, followed by two washes of 5 min in PBTx 0.1 (0.1% Triton X‐100 in PBS, pH 7.4). To prevent nonspecific antibody binding, sections were incubated for 1 h in serum blocking solution (SBS) containing 1 mg/mL bovine serum albumin (BSA, Panreac‐Applichem, A1391), 10% fetal bovine serum (Sigma‐Aldrich, F9665), and PBTx 0.1. Primary antibodies were diluted in SBS and incubated overnight at 4°C. For secondary antibody incubation, sections were washed four times for 10 min in PBTx 0.1 and then incubated with the appropriate secondary antibodies diluted in SBS for 4 h, followed by three washes of 5 min in PBS. Nuclear counterstaining was performed by incubating sections with 4′,6‐diamidino‐2‐phenylindole (DAPI; Sigma‐Aldrich, D9542) diluted 1:1000 in PBS for 10 min, followed by two washes of 5 min in PBS. Sections were mounted on glass slides with coverslips using DAKO Fluorescence Mounting Medium (DAKO, S302380‐2). For LRRK2 immunostaining, antigen retrieval was performed prior to the immunofluorescence or immunohistochemistry protocols. Sections were washed twice for 5 min in 1× PBS and subjected to six cycles of incubation in 0.1 M citrate buffer (monobasic sodium citrate; Sigma‐Aldrich, 71 497) at 90°C for 5 min each, followed by two washes of 5 min in 1× PBS. Horseradish peroxidase (HRP)–3,3′‐diaminobenzidine (DAB)–based immunohistochemistry was also performed on free‐floating sections under conditions similar to those described for immunofluorescence, unless otherwise indicated. Sections were pretreated with 0.4% H_2_O_2_ for 30 min to quench endogenous peroxidase activity. Washing steps and primary antibody incubation were performed as described above. For secondary antibody incubation, sections were washed four times for 10 min in PBTx 0.1 and then incubated with a commercial secondary antibody solution for 2 h, followed by two washes of 5 min in 1× PBS. All subsequent steps were performed under a chemical fume hood due to DAB toxicity. Signal visualization was carried out using a commercial DAB development kit (ScyTeK, ACK500) according to the manufacturer's instructions, except that chromogen exposure time was optimized to 1 min 30 s to enhance contrast. Stained sections were mounted on glass slides with coverslips using 50% glycerol (Sigma‐Aldrich, G7757) in PBS as mounting medium. LRRK2; tyrosine hydroxylase (TH); parvalbumin (PV); choline acetyltransferase (ChAT); Dopamine and cAMP‐regulated phosphoprotein, 32 kDa (DARPP32); glial fibrillary acidic protein (GFAP); and ionized calcium‐binding adapter molecule 1 (IBA1) immunohistological detection was performed as above and described previously (Muñoz‐Manchado et al. 2013; Villadiego, García‐Arriaza, et al. 2023; Villadiego, García‐Swinburn, et al. 2023) using, respectively, anti‐LRRK2 (rabbit polyclonal, 1:500, Abcam, ab133474, RRID:AB_2713963); anti‐TH (rabbit polyclonal, 1:1000, Novus Biological, NB300‐109, RRID:AB_10077691; or mouse monoclonal, 1:1000, Sigma, T1299, RRID:AB_477560); anti‐PV (mouse monoclonal, 1:1000, Sigma, SAB4200545, RRID:AB_2857970); anti‐ChAT (mouse monoclonal, 1:1000, Millipore, MAB5270, RRID:AB_2079753); anti‐DARPP32 (goat polyclonal, 1:100, Santa Cruz, sc‐8384); anti‐GFAP (rabbit polyclonal, 1:500, Dako, Z0334, RRID:AB_10013382); and anti‐IBA1 (rabbit polyclonal, 1:500, Raffer‐Wako, 019–19 741, RRID:AB_839504) and secondary peroxidase‐conjugated antibody kits (NeoBiotech, NB‐23‐00029‐1 and NB‐23‐00030‐1) or fluorescence secondary antibodies (donkey‐anti‐rabbit AlexaFluor488, 1:400, Thermo Fisher Scientific, A‐21206, RRID:AB_2535792; donkey‐anti‐mouse AlexaFluor488, 1:400, Thermo Fisher Scientific, A‐21202, RRID:AB_141607; goat‐anti‐rabbit AlexaFluor568, 1:400, Thermo Fisher Scientific, A‐11036, RRID:AB_10563566; goat‐anti‐mouse AlexaFluor568, 1:400, Thermo Fisher Scientific, A‐11004, RRID:AB_2534072; donkey‐anti‐goat AlexaFluor568, 1:400, Thermo Fisher Scientific, A‐11057, RRID:AB_2534104). For immunofluorescence and immunohistochemistry stainings, negative control sections were processed by omitting the primary antibody while maintaining all other experimental conditions. No specific immunoreactivity was detected in these conditions.

### 2.5 Image Analysis and Stereology

Image acquisition and analysis were performed with light transmission (Olympus, AX70 or Bx61, both with digital refrigerated camera DP72, CellSens v 1.4.1; RRID:SCR_014551) or confocal microscopes and their specific imaging software (Nikon A1R+ or Leica STELLARIS 8 Scan Head, respectively, NIS‐Element AR v 4.30.02; RRID:SCR_014329 and LAS X v4.3.024308; RRID:SCR_013673). For quantitative analyzes, including optical density (OD) measurements and stereological assessments, the experimenter was blinded to the experimental groups during analysis. In all experiments, each mouse was considered an independent biological replicate, and each data point in the graphs represents a single animal. The striatal and SNpc region studied was, respectively, from 1.10 to −0.14 mm and from −2.92 to −3.40 mm respect to Bregma (Paxinos and Franklin 1997). Every fifth section of striatum and midbrain was analyzed, resulting in a total of five sections per region and animal. All measurements were bilateral and averaged per animal. Slides with no uniform staining (e.g., faint stripes within strong staining, or ink‐like blotches) were excluded from analysis.

OD measurements, after TH or GFAP immunohistochemistry, were performed as previously described (Muñoz‐Manchado et al. 2016; Villadiego, Romo‐Madero, et al. 2018) using Fiji v2.3.0 (National Institutes of Health; RRID:SCR_002285). All imaging parameters were kept constant across samples: light condenser off, Nomarski filters off (upper and lower), light intensity set to 7.0, LBD filter engaged, TLD closed, camera lock half‐open, exposure time of 400 μs for TH and 1.2 ms for GFAP, and no digital contrast enhancement. For TH analysis, background correction was performed using the corpus callosum, without TH immunoreactivity. For GFAP analysis, background signal was subtracted using areas of the image devoid of tissue (white background).

Stereological quantification of SNpc TH^+^ neurons was performed using the optical dissector method (West 1993) as previously indicated (Muñoz‐Manchado et al. 2016, 2013; Villadiego, García‐Swinburn, et al. 2023). Reference volumes for each section were outlined under low magnification (4×), and TH^+^ neurons were counted at high magnification (40×) using a 7225 μm^2^ × 20 μm optical dissector. SNpc TH^+^ soma volume was determined by applying the stereological estimator of volume nucleator (Gundersen 1988; Villadiego, García‐Swinburn, et al. 2023) with vertical uniform random sampling (VUR). The stereological quantification of resting and active IBA1^+^ microglia was done as previously stated by the lab (Muñoz‐Manchado et al. 2016; Villadiego, Labrador‐Garrido, et al. 2018; Villadiego, Romo‐Madero, et al. 2018), using a 12427.7 μm^2^ × 20 μm optical dissector. Microglia cell body volume was also measured using the nucleator estimator with VUR, using a threshold of 300 μm^3^ to determine whether microglia were resting (< 300 μm^3^) or active (> 300 μm^3^). All stereological procedures were performed using the New CAST system (Visiopharm; RRID:SCR_014570) and, in all cases, a guard volume of 5 μm was applied to avoid artifacts on the cut surface of the sections. During all stereological procedures, illumination parameters and exposure times were software‐defined and uniformly applied to all samples.

### 2.6 Measurement of Striatal Dopamine and Metabolite Levels

Striatal dopamine (DA), 3,4‐dihydroxyphenylacetic acid (DOPAC), and homovanillic acid (HVA) levels were determined in untreated (young adult or aged mice), 20 mg/kg or 5 mg/kg MPTP‐treated mice and their respective saline‐treated controls. In all cases, the experimenter was blinded to the experimental groups during analysis. Striatal tissue was sonicated in 200 μL of a solution containing 0.1 M HClO_4_, 0.02% EDTA, and 1% ethanol (Sigma‐Aldrich, respectively 244 252, E9884, and 24103) using a Branson Sonifier 150D (power setting 2) in 1.5 mL tubes. Sonication consisted of six 10 s pulses separated by 10 s intervals while samples were kept on ice to prevent heating. The homogenate was centrifuged at 16000 *g* for 10 min at 4°C. The supernatant was filtered through a 30 000 Da molecular mass exclusion membrane (Millipore, UFC503096) by centrifugation at 16000 *g* for 30 min.

Striatal samples from untreated (young adult or aged) mice, 20 mg/kg MPTP‐treated mice, and their corresponding saline‐treated controls were analyzed by high performance liquid chromatography with electrochemical detection (HPLC‐ECD) at IBiS, as previously described (Villadiego, Romo‐Madero, et al. 2018; Villadiego, García‐Swinburn, et al. 2023). Briefly, filtered supernatants were injected into an HPLC pump system (ALEXYS 100, Antec) equipped with a 3 μm C‐18 column (ALB‐215, Antec). Catecholamines were detected electrochemically using a glassy carbon electrode and an ISAAC reference electrode (DECADE II, VT‐03 flow cell, Antec). Chromatography was performed at 35°C with a flow rate of 200 μL/min. The HPLC mobile phase consisted of 50 mM H_3_PO_4_ (Sigma‐Aldrich, 49 685), 50 mM citric acid (Sigma‐Aldrich, 251 275), 8 mM KCl (Sigma‐Aldrich, P4504), 0.1 mM EDTA (Sigma‐Aldrich, E9884), 0.4 mg/mL octane‐sulphonic acid (Sigma‐Aldrich, RDD032), and 3% (v/v) methanol (Sigma‐Aldrich, 34860), adjusted to pH 4.15. The mobile phase was filtered through a 0.22 μm membrane and degassed for 10 min before use. Calibration curves were prepared over the range of 7.7–766 μg/L for DA (Sigma‐Aldrich, H8502), 8.4–841 μg/L for DOPAC (Sigma‐Aldrich, 850 217), and 9.1–911 μg/L for HVA (Sigma‐Aldrich, H1252) by linear regression (*R* ^2^ > 0.96). DA, DOPAC, and HVA concentrations were expressed as μg/L.

Striatal samples from 5 mg/kg MPTP‐treated mice and their respective saline‐treated controls were analyzed by ultra‐performance liquid chromatography coupled to tandem mass spectrometry (UPLC–MS/MS) at the General Microanalysis Research Service (CITIUS, University of Seville). Analyzes were performed using a Waters Xevo TQ‐XS triple quadrupole mass spectrometer coupled to an ACQUITY UPLC BEH C18 column maintained at 40°C. Chromatographic separation was achieved using a gradient of water containing 0.05% formic acid (Sigma‐Aldrich, F0507) and methanol (Sigma‐Aldrich, 34 860) at a flow rate of 0.3 mL/min, with a total run time of 8 min and an injection volume of 10 μL. Quantification was performed in positive electrospray ionization mode using multiple reaction monitoring (MRM). Calibration curves were prepared over the range of 0.1–500 μg/L for DA (Sigma‐Aldrich, H8502), DOPAC (Sigma‐Aldrich, 850 217), and HVA (Sigma‐Aldrich, H1252) by linear regression (*R* ^2^ ≥ 0.99). The limit of detection was established at 1 μg/L. DA, DOPAC, and HVA concentrations were expressed as μg/L. No internal standard was included for either HPLC‐ECD or UPLC–MS/MS analyzes. Quantification was based on external calibration curves. HPLC‐ECD and UPLC–MS/MS measurements were obtained as single injections without technical replicates.

Pellets obtained after centrifugation were resuspended in 200 μL of 0.1 M NaOH (Sigma‐Aldrich, 221 465), and protein concentrations were determined using the Bradford assay (Bio‐Rad, 500–0006) from the mean of technical duplicates. Absorbance at 595 nm was measured using a spectrophotometer (Multiskan Spectrum, Thermo, 51118500). Calibration curves were prepared using BSA standards (PanReac AppliChem, A1391) ranging from 0.74 to 3.70 μg/μL by linear regression (*R* ^2^ > 0.98). No samples were below the limit of detection for catecholamine or protein quantification. Catecholamine concentrations were normalized to total protein content (ng/mg protein) and expressed as a percentage of untreated young WT mice, or WT saline‐treated mice, depending on the experimental series.

### 2.7 RNA Isolation, RT‐qPCR and Digital PCR

For untreated WT and hLRRK2*G2019S mice, either young adults or aged, total RNA was isolated using TRIzol Reagent (Thermo Fisher Scientific, 15596026) according to the manufacturer's instructions. Briefly, samples were homogenized in TRIzol Reagent using a rotor–stator homogenizer (PRO Scientific Inc., 03436) and adjusted to a final volume of 1 mL. Homogenates were centrifuged (15.000 *g*, 10 min) to remove debris, and the supernatant was extracted twice with chloroform (Sigma‐Aldrich, C2432), followed by centrifugation (10.000 *g*, 10 min) to separate the aqueous phase. RNA was precipitated by adding an equal volume of isopropanol (Sigma‐Aldrich, I9516) and incubating for 10 min at room temperature before centrifugation (15 000 *g*, 10 min). The RNA pellet was washed with 75% ethanol prepared from absolute ethanol (Sigma‐Aldrich, E7023), air‐dried, and resuspended in DNase/RNase‐free water (Sigma‐Aldrich, W4502). RNA concentration and purity were determined using a NanoDrop 2000 spectrophotometer (ThermoFisher Scientific, ND 2000). First‐strand cDNA was synthesized from 1 μg of total RNA using the QuantiTect Reverse Transcription Kit (Quiagen, 205 311), according to the manufacturer's specifications. Relative mRNA expression levels were calculated, using the $2^{-\Delta \Delta C_{t}}$ method with the young adult group used as the calibrator, by quantitative real‐time PCR (RT‐qPCR) on a ViiA 7 real‐time PCR System (Thermo Fisher Scientific) and the following TaqMan Gene Expression Assays (Thermo Fisher Scientific): *interleukin 1 beta* (*Il1b*, Mm00434228_m1), *interleukin 6* (*Il6*, Mm00446190_m1), *transforming growth factor beta 1* (*Tgfb1*, Mm01178820_m1), *brain‐derived neurotrophic factor* (*Bdnf*, Mm04230607_s1), *tumor necrosis factor* (*Tnf*, Mm00443258_m1), *interferon gamma* (*Ifng*, Mm01168134_m1), *interleukin 10* (*Il10*, Mm01288386_m1), interleukin 4 (*Il4*, Mm00445259_m1), and *glial cell line‐derived neurotrophic factor* (*Gdnf*, Mm00599849_m1). Reactions were performed under the cycling conditions recommended by the manufacturer. Expression levels were normalized to *peptidylprolyl isomerase A (Ppia*, Mm02342430_g1; Thermo Fisher Scientific), which was used as the endogenous reference gene to correct for variations in RNA input. All samples were analyzed in technical triplicate.

For WT and hLRRK2‐G2019S mice treated with MPTP (20 or 5 mg/kg) and their respective saline‐treated controls, RNA was isolated from microdissected striatal (from +1.10 to −0.14 mm relative to Bregma) and ventral mesencephalic (from −2.92 to −3.40 mm relative to Bregma) tissue obtained from 10 to 20 coronal brain sections (4% PFA fixed, 30 μm of thickness), according to the mouse brain atlas (Paxinos and Franklin 1997). Microdissection was performed under a stereoscopic microscope (Olympus SZX16). Total RNA was extracted using the RecoverAll Total Nucleic Acid Isolation Kit (Invitrogen, Thermo Fisher Scientific, AM1975) according to the manufacturer's instructions. RNA concentration and purity were assessed as described above. Transcript levels of the previously described target genes were quantified using the corresponding TaqMan Gene Expression Assays and digital PCR (dPCR) QuantStudio 7 Flex platform (Thermo Fisher Scientific). Reverse transcription and dPCR amplification were performed using a one‐step workflow directly on dPCR platform (SAIBIS‐IBiS), using QuantStudio Absolute Q One‐Step RT‐PCR Mix (Applied Biosystems, A55146) with the following protocol: RNA‐RT 55°C for 10 min, preheat 96°C for 10 min, followed by 40 cycles of step one, 96°C for 5 s and step two 60°C for 10 s. Absolute transcript quantification was determined as copies per ng of total RNA input, values were normalized to the mean of the genotype‐matched saline group, and expressed as fold change.

### 2.8 Statistical Analysis

The number of mice analyzed in each experimental group is indicated in the plot as individual scatter points, and the statistical tests applied are specified in the statistical report available in the Supporting Information. Data are presented as the mean ± SEM. In all cases, normality [Anderson‐Darling (A2), D'Agostino‐Pearson omnibus (K2), Shapiro–Wilk (W) and Kolmogorov–Smirnov (distance)] and equal variance (*F* test for two‐groups; and Brown‐Forsythe or Bartlett's tests for multiple‐groups comparisons) tests were performed; when passed, unpaired *t*‐tests (for two‐group comparisons) or analysis of variance (ANOVA) with Dunnett post hoc analysis (for multiple groups) were carried out. When homoscedasticity tests failed, Welch's *t*‐test (two groups) or Brown‐Forsythe and Welch ANOVA tests (multiple groups) were used. In cases where normality test failed, the nonparametric *U*‐Mann Whitney (two groups) or Kruskal–Wallis *H* test with post hoc Dunn's test (multiple groups) was performed. All statistical analyzes were conducted using Prism v.8.0 (GraphPad Software, RRID:SCR_002798). Statistical significance was defined as *p* < 0.05. All *p*‐values are presented in the figure panels, with significant results highlighted in bold. A detailed statistical report is available in the Supporting Information.

### 2.9 Use of Artificial Intelligence‐Assisted Tools

ChatGPT (OpenAI; GPT‐5.5) was used in order to review and correct the wording and grammar of the manuscript. In addition, ChatGPT was used as an independent verification tool to check the correspondence between the statistical results reported in the figures and Supporting Information and the outputs generated by Prism v.8.0 (GraphPad Software, RRID:SCR_002798). This verification was performed as a double‐check procedure, whereby all statistical values were independently reviewed by both the authors and the AI‐assisted tool. All final decisions regarding data analysis, statistical reporting, interpretation of the results, and manuscript content were made exclusively by the authors, who verified the accuracy of all information included in the final version of the manuscript.

## 3 Results

### 3.1 Characterization of LRRK2 Expression in hLRRK2*G2019S Mice

**FIGURE 1:**
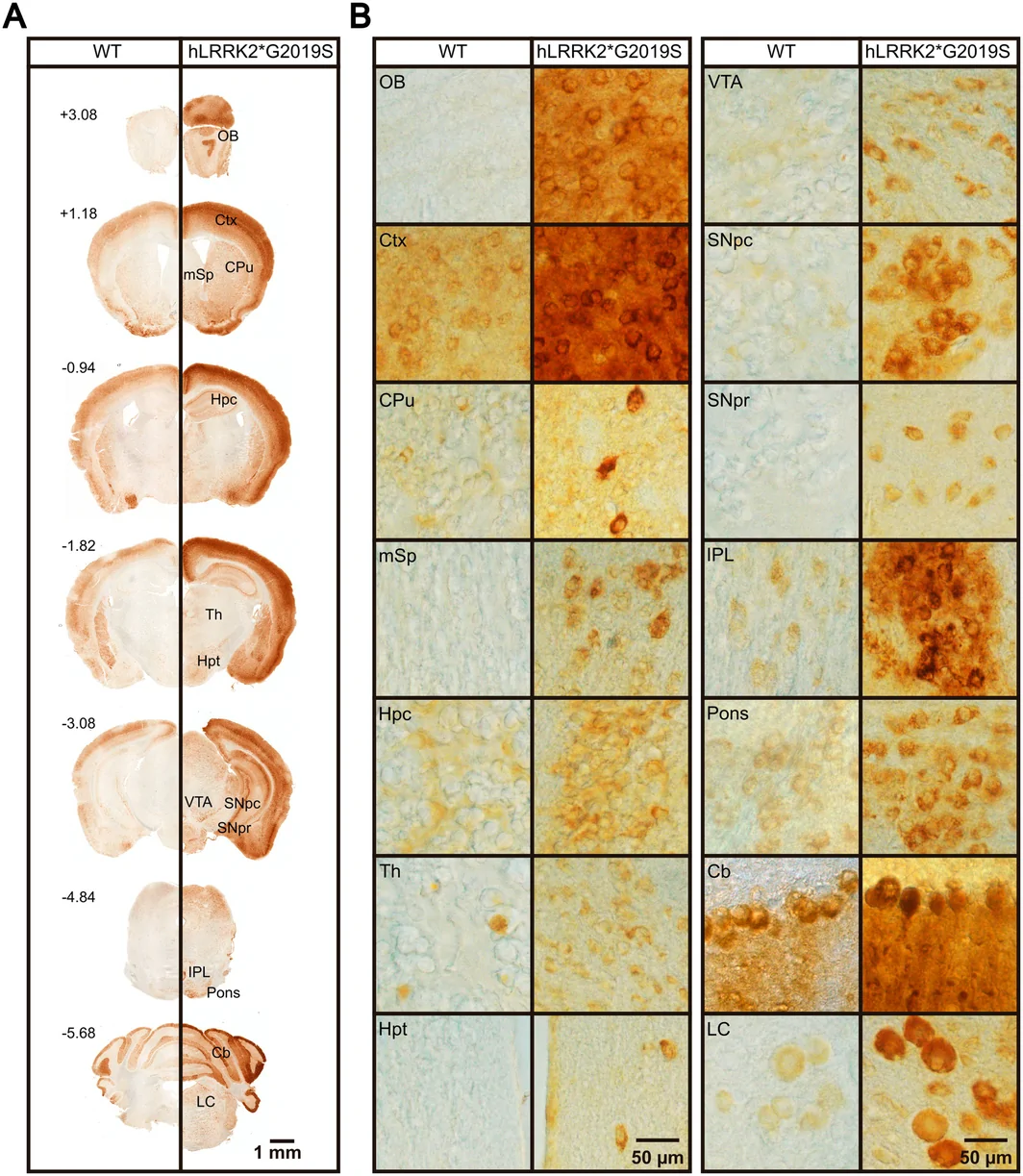
Overexpression of LRRK2 in hLRRK2*G2019S mouse model. (A) Rostro‐caudal series of low‐magnification brightfield images of mice brain coronal sections stained against LRRK2, showing basal expression in WT mice (left) and overexpression in the hLRRK2*G2019S mice (right). Distance from Bregma (mm) are indicated. Regions marked are: Olfactory bulb (OB), cortex (Ctx), caudate putamen (CPu), medial septum nucleus (mSp), hippocampus (Hpc), hypothalamus (Hpt), thalamus (Th), ventral tegmental area (VTA), substantia nigra pars compacta (SNpc), substantia nigra pars reticulata (SNpr), pons, interpeduncular lateral nucleus (IPL), locus coeruleus (LC) and cerebellar cortex (Cb). (B) Series of high magnification brightfield images of cells in regions described in A.

The hLRRK2*G2019S mouse model was generated using 188 kb bacterial artificial chromosome (BAC) containing the full human LRRK2 gene with the G2019S mutation, including its native promoter and regulatory elements (Baptista et al. 2013; “JAX 018785 ‐ Strain Details”). To confirm transgene overexpression, LRRK2 immunohistochemistry was conducted in coronal brain sections of WT and hLRRK2*G2019S mice, using an antibody that detects both the endogenous and mutant LRRK2*G2019S proteins (Longo et al. 2017; Xenias et al. 2022). Given that ubiquitous and constitutive expression of LRRK2 has been reported in the mammalian brain (Biskup et al. 2006; West et al. 2014), representative regions of the brain were examined to assess overexpression (Figure 1). The results revealed markedly stronger LRRK2 staining in hLRRK2*G2019S mice compared to WT controls, where the staining ranged from moderate to faint. Specifically, the anterior olfactory area, cortex, caudate‐putamen (CPu, hereafter also referred to as the striatum), and medial septum showed increased LRRK2 expression in the hLRRK2*G2019S mice. The hippocampus, mesencephalon, pons, and lateral interpeduncular nuclei also demonstrated elevated LRRK2 levels in transgenic mice. In particular, the locus coeruleus, the internal granular layer, and Purkinje cells of the cerebellar cortex exhibited significant overexpression in hLRRK2*G2019S mice, compared to weak signals found in WT mice. These findings confirm the widespread overexpression of the hLRRK2 G2019S protein in the transgenic mouse brain, supporting its utility to study the functional consequences of hLRRK2 G2019S expression in the mammalian brain.

**FIGURE 2:**
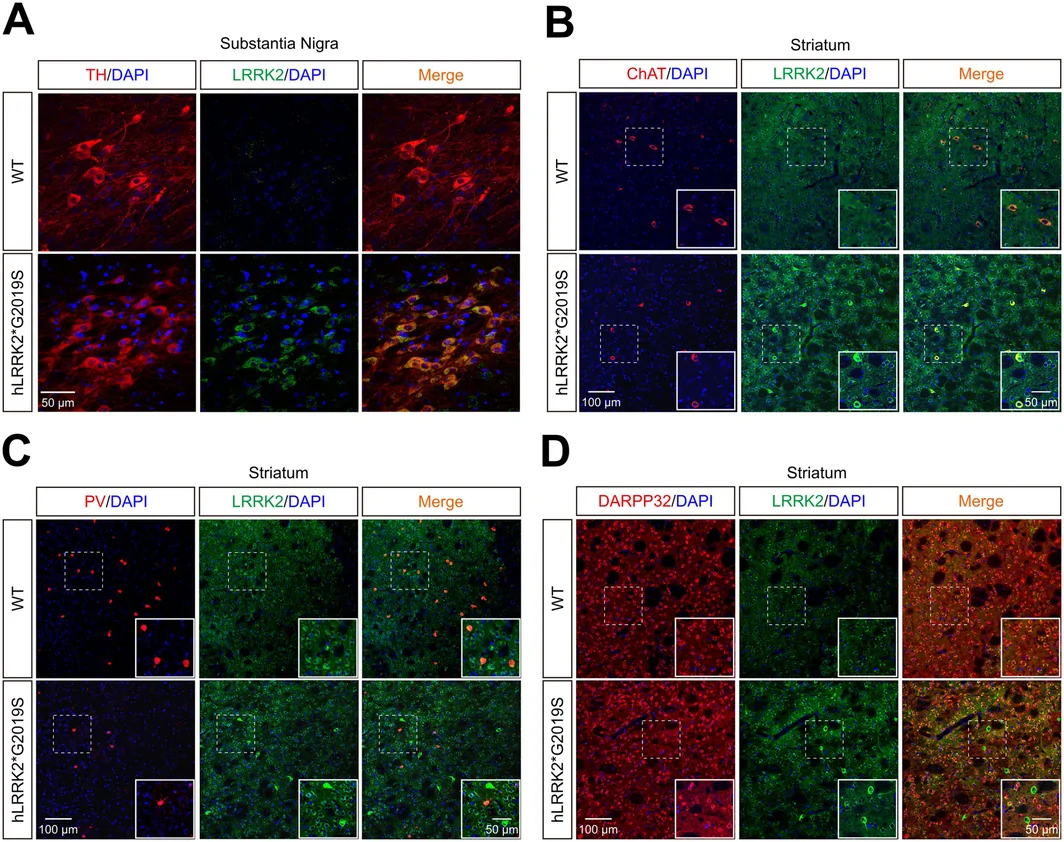
Identity of nigrostriatal LRRK2‐overexpressing cells in hLRRK2*G2019S mice. (A) Confocal images of dopaminergic (TH^+^; red) substantia nigra neurons, from hLRRK2*G2019S and WT mice, exhibiting the overexpression of the hLRRK2 G2019S transgene (LRRK2^+^; green). (B–D) Confocal images of striatal cholinergic interneurons (B, ChAT^+^; red), fast‐spiking interneurons (C, PV^+^; red) and medium spiny neurons (D, DARPP32^+^; red), showing colocalization of high LRRK2 signal with ChAT^+^ neurons, without coinciding with baseline LRRK2 expressing cells (B). Note that baseline LRRK2 signal is observed in PV^+^ and DARRP32^+^ neurons (red, C, D), without colocalizing with high LRRK2 signal. Nuclei were counterstained with DAPI (blue).

Regarding the transgene expression in the nigrostriatal pathway, LRRK2 immunohistochemistry in the SNpc revealed a staining pattern that suggest a significant overexpression of the hLRRK2 G2019S protein in dopaminergic neurons (Figure 1A,B, CPu, VTA, SNpc). To confirm that, high resolution confocal microscopy analysis was performed combining LRRK2 and TH immunofluorescence in both WT and hLRRK2*G2019S mice. As shown in Figure 2A, high LRRK2 staining observed in hLRRK2*G2019S mice colocalize within dopaminergic TH^+^ SNpc neurons, indicating the transgene overexpression in these dopaminergic neurons. The analysis of LRRK2 immunohistochemistry in the striatum showed a stronger basal LRRK2 staining in hLRRK2*G2019S mice compared to WT controls, but also a distinct and pronounced overexpression of LRRK2 in a subset of large cells dispersed throughout the striatum of transgenic mice (Figures 1B and 2B–D). Given the relevance of striatal neurons in PD pathology (Shen et al. 2022), and the relevance of striatal fast‐spiking and cholinergic interneurons in motor control (Gritton et al. 2019; McKinley et al. 2019; Tanimura et al. 2018), we sought to identify the specific population of striatal neurons responsible for this elevated transgene expression. To this end, confocal microscopy studies were also carried out in WT and hLRRK2*G2019S mice, combining LRRK2 immunofluorescence with specific makers of cholinergic interneurons (ChAT^+^), fast‐spiking (parvalbumin^+^, PV^+^), or medium spiny neurons (DARPP32^+^, as the main striatal neuronal population). Intense LRRK2 cellular staining was specifically found within ChAT^+^ cells of transgenic mice (Figure 2B), confirming the hLRRK2G2019S overexpression in cholinergic interneurons of hLRRK2*G2019S mice. In contrast, medium spiny neurons‐DARPP32^+^ or fast‐spiking‐PV^+^ neurons from hLRRK2*G2019S mice only exhibited baseline LRRK2 immunofluorescence levels, comparable to those observed in WT mice (Figure 2C,D).

### 3.2 Analysis of the Nigrostriatal Pathway of Young and Aged hLRRK2*G2019S Mice

**FIGURE 3:**
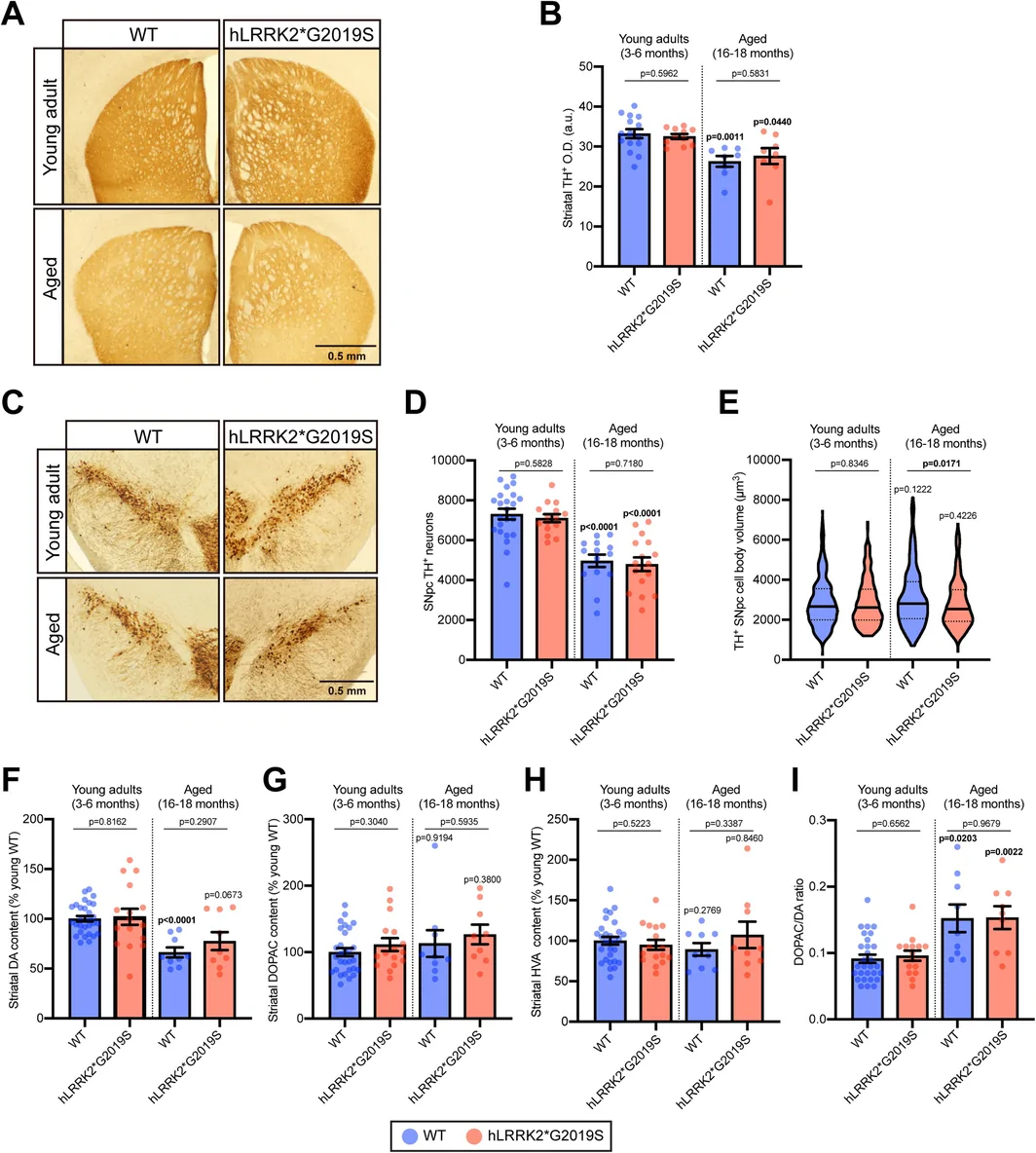
Histological and neurochemical analysis of the nigrostriatal pathway in hLRRK2*G2019S mice. (A) Representative images of striatal coronal sections, immunostained against TH, of young and aged WT and hLRRK2*G2019S mice. (B) Analysis of striatal dopaminergic innervation by TH^+^ O.D. measurements. (C) Representative images of mesencephalic coronal sections, after TH immunohistochemistry, from the previously described experimental groups. (D, E) Stereological quantification of the number (D) and the neuronal cell body volume (E) of SNpc TH^+^ neurons. (F–I) Striatal content of DA (F), DOPAC (G), HVA (H), and DA metabolic rate expressed as DOPAC/DA ratio (I). *p*‐values above connecting lines indicate pairwise statistical comparisons. Individual *p*‐values above each bar indicate comparisons with the young controls of the corresponding genotype.

The integrity and features of the nigrostriatal pathway was assessed in young adults (3–6 months) or aged (16–18 months) hLRRK2*G2019S mice, compared to age‐matched WT controls, by analysis of coronal striatal and mesencephalic sections after TH immunohistochemistry. In the striatum, no differences were observed in dopaminergic striatal innervation, measured by TH OD, between hLRRK2*G2019S mice and WT controls, neither in young adults nor aged animals (Figure 3A,B). Similarly, no significant differences were detected between these experimental groups in the stereological estimation of SNpc TH^+^ neurons (Figure 3C,D). However, the stereological analysis of the neuronal soma volume revealed a reduction of the volume of dopaminergic SNpc neurons from aged hLRRK2*G2019S mice when compared with age‐matched WT controls (Figure 3E). Striatal DA and metabolites content (DOPAC and HVA) were also analyzed by HPLC, without significant variations between hLRRK2*G2019S mice and age‐matched WT controls (Figure 3F–H). Furthermore, the DOPAC/DA ratio was calculated as readout of DA turnover in dopaminergic terminals, being comparable between both genotypes, either in young adults or aged mice (Figure 3I). Overall, the histological and neurochemical analyzes revealed comparable levels of striatal dopaminergic innervation, DA content and SNpc dopaminergic neurons between WT and hLRRK2*G2019S mice across both young and aged cohorts, except for a significant reduction in SNpc neuron soma volume in aged hLRRK2*G2019S mice. In addition, consistent with previous reports (Noda et al. 2020; Yue et al. 2015), a similar age‐related neurodegeneration was observed between WT and hLRRK2*G2019S mice, which is evident as reduction in dopaminergic striatal innervation (Figure 3A,B), number of SNpc TH^+^ neurons (Figure 3C,D), striatal DA content (Figure 3F) and increased striatal DOPAC/DA ratio (Figure 3I).

### 3.3 Behavioral Analysis

**FIGURE 4:**
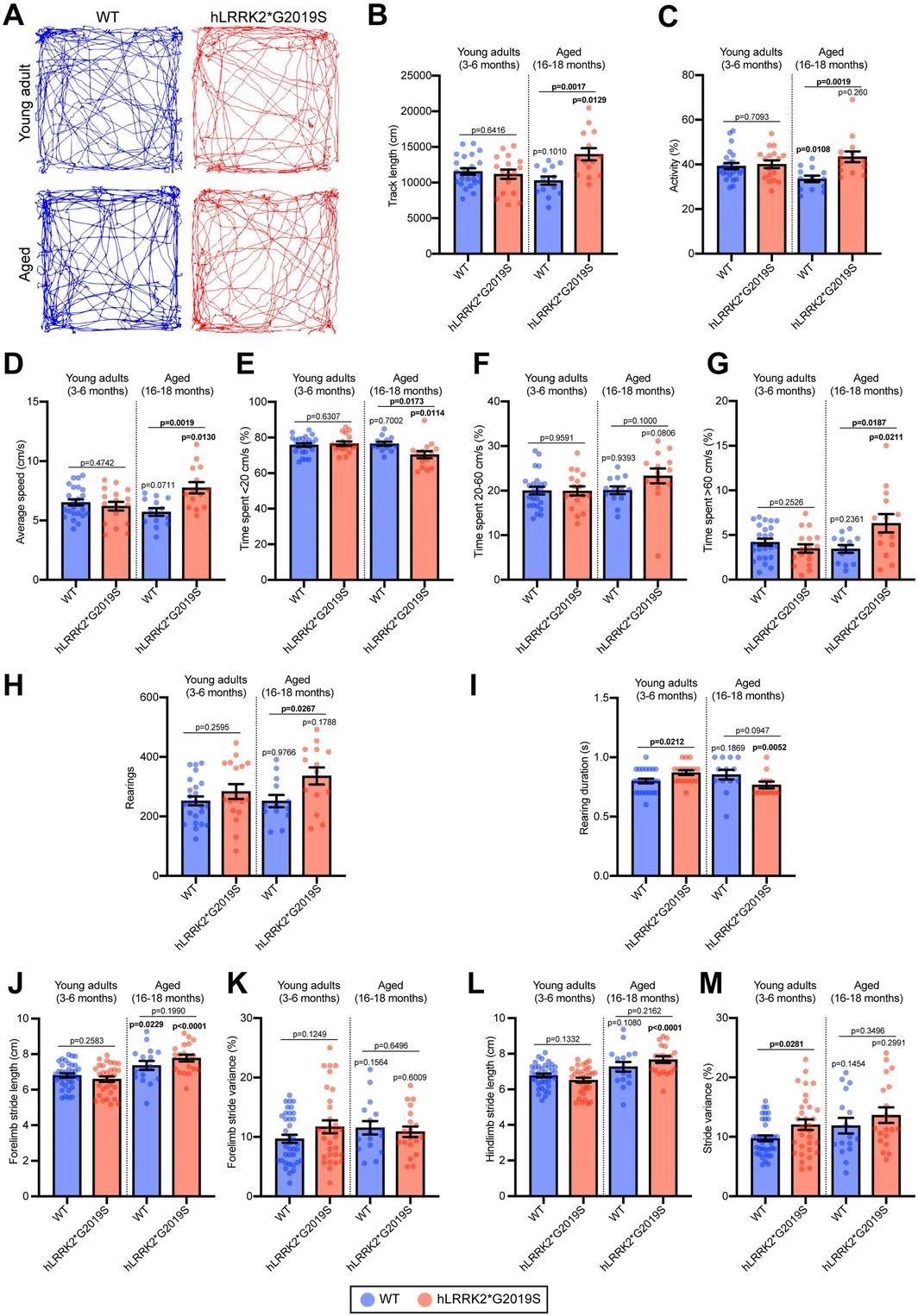
Behavioral analysis of hLRRK2*G2019S mice. (A) Openfield tracking of young and aged WT and hLRRK2*G2019S mice. (B, C) Data of total track length (B) and activity (C). (D–G) Velocity data, showing the average speed (D) and the percentage of time spent at: Low velocities (< 20 m/s; E), intermediate velocities (20–60 m/s; F), and high velocities (> 60 m/s; G). (H, I) Vertical activity in the openfield test, indicating the rearings count (H) and rearings duration (I). (J–M) Footprint analysis of the previously described experimental groups, forelimb stride length (J) and variance (K) and hindlimbs stride length (L) and variance (M) are shown. *p*‐values above connecting lines indicate pairwise statistical comparisons. Individual *p*‐values above each bar indicate comparisons with the young controls of the corresponding genotype.

Next, motor function was evaluated in hLRRK2*G2019S mice and age‐matched WT, using specific test of motor activity and gait coordination. Openfield test was used to assess overall activity. In young adult mice, no significant differences were found between WT and transgenic mice in track length (Figure 4A,B), activity (Figure 4C), average speed (Figure 4D), or time spent in low (< 20 cm/s), medium (20–60 cm/s), or high (> 60 cm/s) velocity ranges (Figure 4E–G). Similarly, no differences were detected in rearing counts (Figure 4H) between these two experimental groups, but a slight increase in rearing duration was detected in young adult hLRRK2*G2019S mice respect to WT controls (Figure 4I). In contrast, the analysis of the aged cohort revealed a hyperactive phenotype of aged hLRRK2*G2019S mice, depicted by significantly higher track length, activity, average speed and time spent at high velocity, with a corresponding decrease in time spent at low velocity range (Figure 4A–E). These aged hLRRK2*G2019S mice also displayed elevated rearing counts, indicating heightened vertical activity compared to their age‐matched WT controls (Figure 4H).

Gait coordination was further evaluated using the footprint test. In young adult mice, no significant differences were observed in stride length, for either forelimbs or hindlimbs, between WT and hLRRK2*G2019S groups. However, a trend toward increased stride variance in the forelimbs (WT: 9.69 ± 0.69 vs. hLRRK2*G2019S: 11.70 ± 1.09; *p* = 0.1249; Figure 4K) and a significant increase in hindlimb variance (Figure 4M) were observed in hLRRK2*G2019S mice, indicating impaired gait coordination in young adult transgenic mice. In the aged cohort, both genotypes showed age‐related declines in gait coordination, with increased stride length compared to their younger counterparts (Figure 4J,L).

### 3.4 Glial Reaction of Young and Aged hLRRK2*G2019S Mice

**FIGURE 5:**
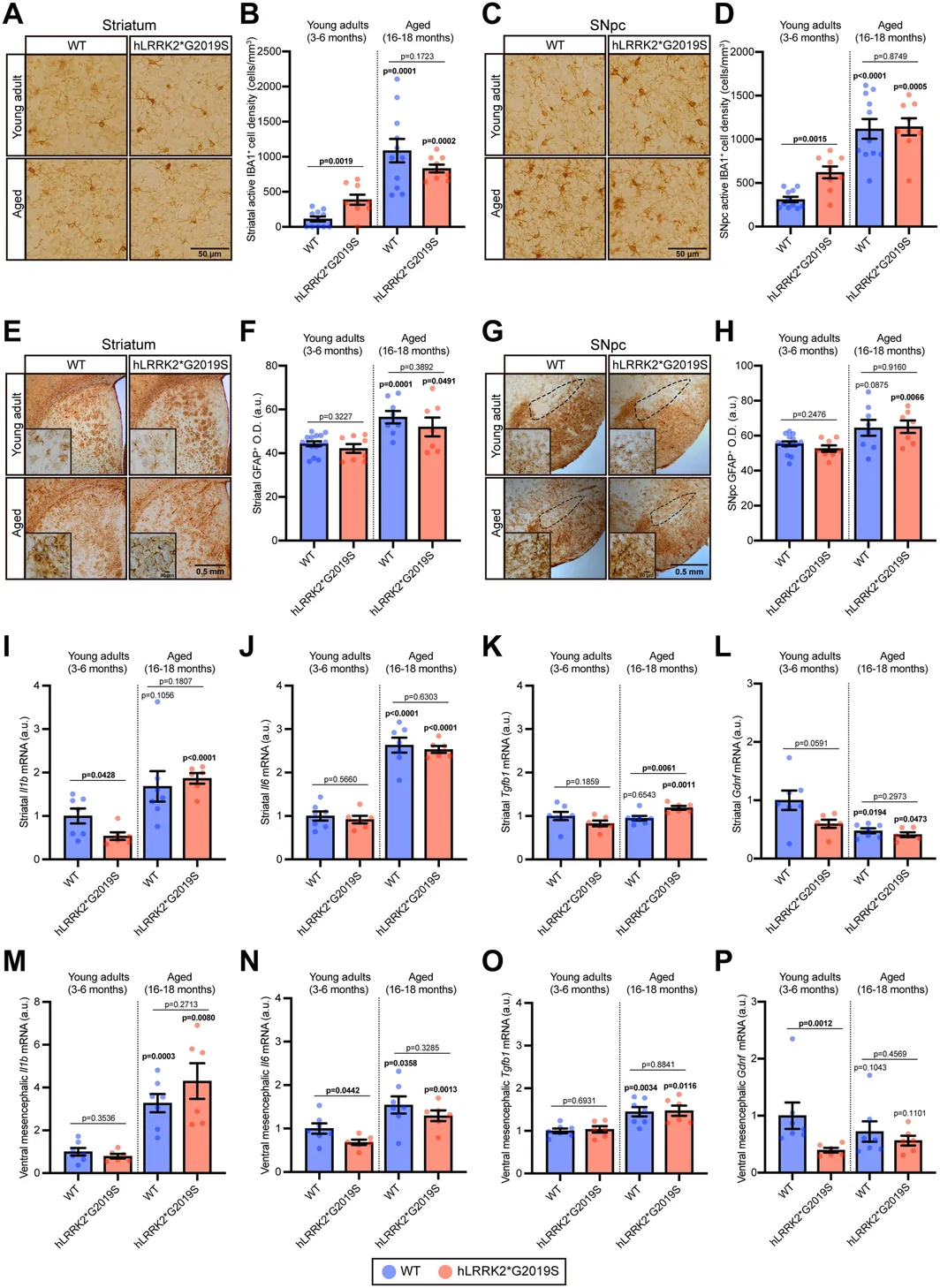
Nigrostriatal glial response of hLRRK2*G2019S mice. (A–D) Nigrostriatal microglial activation of young adult and aged WT and hLRRK2*G2019S mice, displaying high magnification images of Iba1^+^ cells together with the stereological quantification of active microglia, in the striatum (A, B) and SNpc (C, D). (E–H) Astroglial response in the nigrostriatal pathway, of previously indicated experimental groups, showing representative images of coronal sections, after GFAP immunohistochemistry, and corresponding analysis of GFAP^+^ O.D. in the striatum (E, F) and SNpc (G, H). Insets in panels E and G show representative high magnification images of GFAP^+^ staining in the striatum and SNpc from the different experimental groups. Dashed lines in panel G delineate the SNpc. (I–P) Relative mRNA expression of *Il1b* (I, M), *Il6* (J, N), *Tgfb1* (K, O) and *Gdnf* (L, P) in the striatum and ventral mesencephalon, normalized to the mean value of the young adult group. *p*‐values above connecting lines indicate pairwise statistical comparisons. Individual *p*‐values above each bar indicate comparisons with the young controls of the corresponding genotype.

Glial activation, including both microglial and astrocytic responses, is a key contributor to the pathophysiology of PD. Although recent works have studied the involvement of LRRK2 in the neuroimmune response associated to PD (Kim et al. 2020; Zhang et al. 2022; Scheiblich et al. 2024), the specific glial response within the nigrostriatal pathway in LRRK2 mouse models remains poorly characterized. To address this, microglial and astrocytic reactions were studied in young adult and aged hLRRK2*G2019S mice and their respective WT controls, both in striatum and SNpc. To evaluate microglial activation, identified by morphological changes such as cell body enlargement and process retraction (Kettenmann et al. 2011), we performed immunostaining with the microglial marker Iba1 and subsequent stereological analysis of cell morphology and volume following established protocols (Muñoz‐Manchado et al. 2016; Villadiego, Labrador‐Garrido, et al. 2018; Villadiego, Romo‐Madero, et al. 2018). Young adult hLRRK2*G2019S mice exhibited a significant increase in active Iba1^+^ microglia compared to WT (Figure 5A–D). Whereas, in the aged cohort, both genotypes showed increased microgliosis, without any sign of exacerbation observed in transgenic mice relative to their age‐matched WT counterparts (Figure 5A–D). To study the astrogliosis associated to parkinsonism, the astrocytic marker GFAP^+^ immunoreactivity was measured as previously described (Muñoz‐Manchado et al. 2016; Villadiego, Romo‐Madero, et al. 2018). In young adult mice, no significant differences in GFAP^+^ staining were found between hLRRK2*G2019S and WT groups, neither in striatum nor SNpc (Figure 5E–H). Regarding the aged cohort, there was observed an age‐related increase in the astroglial GFAP^+^ response in the nigrostriatal pathway of both WT (~30% in the striatum and ~15% in SNpc) and hLRRK2G2019S (~25% in striatum and SNpc), without significant differences between the two genotypes (Figure 5E–H).

To further characterize the neuroimmune profile observed in hLRRK2*G2019S mice, the mRNA expression levels of different pro‐ (*Il1b, Il6, Tnf* and *Ifng*) and anti‐inflammatory cytokines (*Tgfb1, Il10* and *Il4*), as well as neurotrophic factors (*Gdnf* and *Bdnf*), was analyzed in the striatum and ventral mesencephalon (Figure 5I–P and Figure S1). Young adult hLRRK2*G2019S mice showed reduced mRNA expression of *Il1b* in the striatum (Figure 5I) and *Il6* in the ventral mesencephalon (Figure 5N), whereas *Tnf* mRNA were increased in the ventral mesencephalon (Figure S1F). Also, *Gdnf* expression showed a tendency to decrease in the striatum (Figure 5L) and was significantly reduced in the ventral mesencephalon (Figure 5P) compared to age‐matched WT controls. In the aged cohort, both genotypes exhibited a marked increase in the mRNA expression of the pro‐inflammatory cytokines *Il1b, Il6*, and *Tnf* (Figure 5I,J,M,N and Figure S1A,F). In addition, both aged WT and hLRRK2*G2019S mice displayed increased *Tgfb1* mRNA expression in the ventral mesencephalon (Figure 5O), a trend toward reduced striatal *Il4* mRNA expression (Figure S1D), reduced *Gdnf* mRNA expression in the striatum (Figure 5L) and increased *Bdnf* mRNA expression, both in the striatum and ventral mesencephalon (Figure S1E,J). Notably, aged hLRRK2*G2019S mice showed increased striatal expression of the anti‐inflammatory cytokines *Tgfb1* and *Il10* (Figure 5K and Figure S1C) compared to aged WT controls.

Overall, these experiments indicate a complex neuroimmune profile associated to hLRRK2 G2019S mutation. The morphological analysis revealed accelerated microglial reactivity during early adulthood, without differences in astrogliosis, whereas age‐related morphological glial alterations were comparable between genotypes. At the molecular level, hLRRK2‐G2019S mice exhibited region‐ and age‐dependent alterations in cytokine and neurotrophic factor expression, highlighting the complexity of the neuroimmune profile associated with this mutation within the nigrostriatal pathway.

### 3.5 Susceptibility of hLRRK2*G2019S Mice to Chronic MPTP‐Induced Neurodegeneration

**FIGURE 6:**
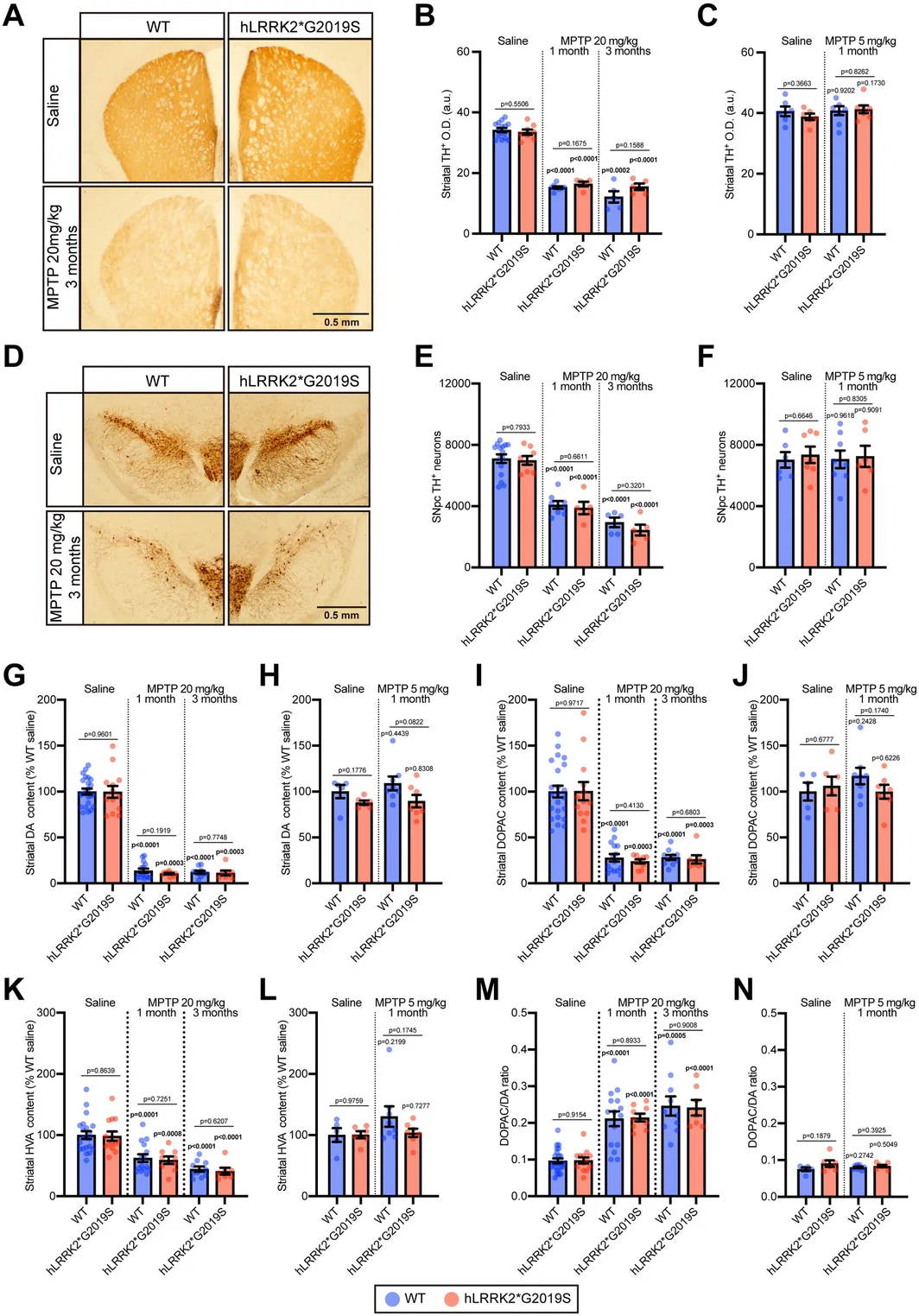
Histological and neurochemical analysis of the nigrostriatal pathway of WT and hLRRK2*G2019S mice chronically treated with MPTP. (A) Representative striatal coronal sections, immunostained for TH, from WT and hLRRK2*G2019S mice treated with saline or MPTP 20 mg/kg for 3 months. (B, C) Analysis of striatal dopaminergic innervation by TH^+^ O.D. measurements from WT and hLRRK2*G2019S mice subjected to the standard chronic MPTP treatment, 20 mg/kg for 1 or 3 months (B); or the sub‐threshold MPTP treatment, 5 mg/kg for 1 month (C). (D) Representative images of mesencephalic coronal sections, after TH immunohistochemistry, from mice described in A. (E, F) Stereological quantification of TH^+^ SNpc neurons of the experimental groups described in B and C. (G–N) Striatal content of DA (G, H), DOPAC (I, J), HVA (K, L), and DA metabolic rate by DOPAC/DA ratio (M, N) from the groups indicated before. *p*‐values above connecting lines indicate pairwise statistical comparisons. Individual *p*‐values above each bar indicate comparisons with the saline controls of the corresponding genotype.

MPTP, via its active metabolite selective for dopaminergic neurons MPP^+^, is a mitochondrial complex I inhibitor, that has been well established to induce dopaminergic neurodegeneration and parkinsonism in mouse and primates (Langston 2017; Henrich et al. 2023). Indeed, parkinsonian LRRK2 mutations have been proposed to impair mitochondrial function (Ilieva et al. 2024; Valderhaug et al. 2024; Rocha et al. 2022). Here, we investigated how hLRRK2 G2019S mutation could affect the susceptibility to long‐term mitochondria complex I inhibition, using a chronic MPTP parkinsonian model that resembles the progressive neurodegeneration of PD (Muñoz‐Manchado et al. 2016). To do so, WT and hLRRK2*G2019S mice were treated with standard doses of MPTP (20 mg/kg, three times per week), or saline solution, during 1 and 3 months. In these mice, a complete histological, neurochemical and behavioral analysis was carried out to study nigrostriatal neurodegeneration, motor function and glial response. The analysis of dopaminergic striatal innervation revealed a significant TH^+^ denervation after 1 and 3 months of MPTP treatment, that was similar both in WT and hLRRK2*G2019S mice respect to their saline treated counterparts (Figure 6A,B). Also, the stereological quantification of SNpc TH^+^ neurons showed a progressive loss of dopaminergic neurons in MPTP‐treated mice, without significant differences between both genotypes (Figure 6D,E). Moreover, the HPLC analysis of the striatal content of DA and its immediate metabolites (DOPAC and HVA) indicated equivalent reductions in DA, DOPAC and HVA content (Figure 6G,I,K), and similar increase in DOPAC/DA ratio (Figure 6M), between WT and hLRRK2*G2019S MPTP‐treated mice, suggesting that both genotypes present similar susceptibility to the nigrostriatal neurodegeneration induced by the toxic treatment. To determine whether the absence of enhanced susceptibility of hLRRK2*G2019S mice to the chronic MPTP treatment could be masked by a ceiling effect, WT and hLRRK2*G2019S mice were exposed to a sub‐threshold MPTP dosage (5 mg/kg, three times per week for 1 month), expected to induce minimal or no dopaminergic damage. The analysis of striatal dopaminergic innervation (Figure 6C) and the number SNpc TH^+^ neurons (Figure 6F) revealed that this sub‐threshold MPTP treatment did not produce significant nigrostriatal dopaminergic damage in either WT or hLRRK2*G2019S mice. Likewise, the study of striatal catecholamines revealed no evidence of dopaminergic damage in either WT or hLRRK2*G2019S mice (Figure 6H,J,L,N), indicating that the expression of hLRRK2 G2019S mutation does not increase susceptibility to chronic MPTP intoxication even under subtoxic conditions.

Motor function was evaluated in mice subjected to neurotoxic treatment, and their saline‐treated controls, using the previously described openfield and footprint test. The openfield test was conducted in WT and hLRRK2*G2019S subjected to chronic MPTP treatment, either at 20 mg/kg for 3 months or at the sub‐threshold dose of 5 mg/kg for 1 month (Figure S2). In mice treated with 20 mg/kg of MPTP for 3 months, no significant differences in track length or average speed were observed among the experimental groups (Figure S2A,B,F). Similar to what was reported on C57BL/6N WT mice (Muñoz‐Manchado et al. 2016), a slight increase in activity was noted in the MPTP‐treated mice, both in WT and hLRRK2*G2019S groups (Figure S2D), which were accompanied by an increase in the time spent at medium velocity rates, and a trend toward reduced time spent at lower or higher velocity rates (Figure S2H,J,L). Also, a tendency to reduce vertical activity, measured as rearing counts and duration, was observed in parkinsonian mice of both genotypes, without significant differences between the WT and hLRRK2*G2019S groups (Figure S2N,P). In agreement with the absence of nigrostriatal damage following the sub‐threshold MPTP treatment (5 mg/kg for 1 month), no significant differences among the experimental groups were observed in any of the openfield parameters analyzed (Figure S2). Furthermore, gait coordination was assessed by the footprint test in WT and hLRRK2*G2019S subjected to chronic MPTP treatment, either at 20 mg/kg, for 1 or 3 months, or at 5 mg/kg for 1 month (Figure S3). As previously observed in untreated WT and transgenic mice (Figure 4K,M), hLRRK2 G2019S mutation induce an increase in the stride variance of saline hLRRK2*G2019S mice (Figure S3C,D,G,H) compared with their saline WT controls, confirming the gait coordination deficits associated with hLRRK2 G2019S expression. In mice treated with 20 mg/kg of MPTP, in agreement with previous reports that showed that chronic MPTP administration increases stride variance (Muñoz‐Manchado et al. 2016), both genotypes exhibited a similar rise in stride variability (Figure S3C,G). Strikingly, after 3 months of neurotoxic treatment, hLRRK2*G2019S mice displayed longer stride lengths than their WT counterparts (Figure S3A,E). In contrast, treatment with the sub‐threshold MPTP dose (5 mg/kg for 1 month) did not produce significant changes in either stride length (Figure S3B,F) or stride variability (Figure S3D,H) in either genotype analyzed. Overall, most of the behavioral analysis performed in WT and hLRRK2*G2019S MPTP‐treated mice indicates that the expression of hLRRK2 G2019S mutation does not modify significantly the motor alterations induced by the neurotoxic treatment.

**FIGURE 7:**
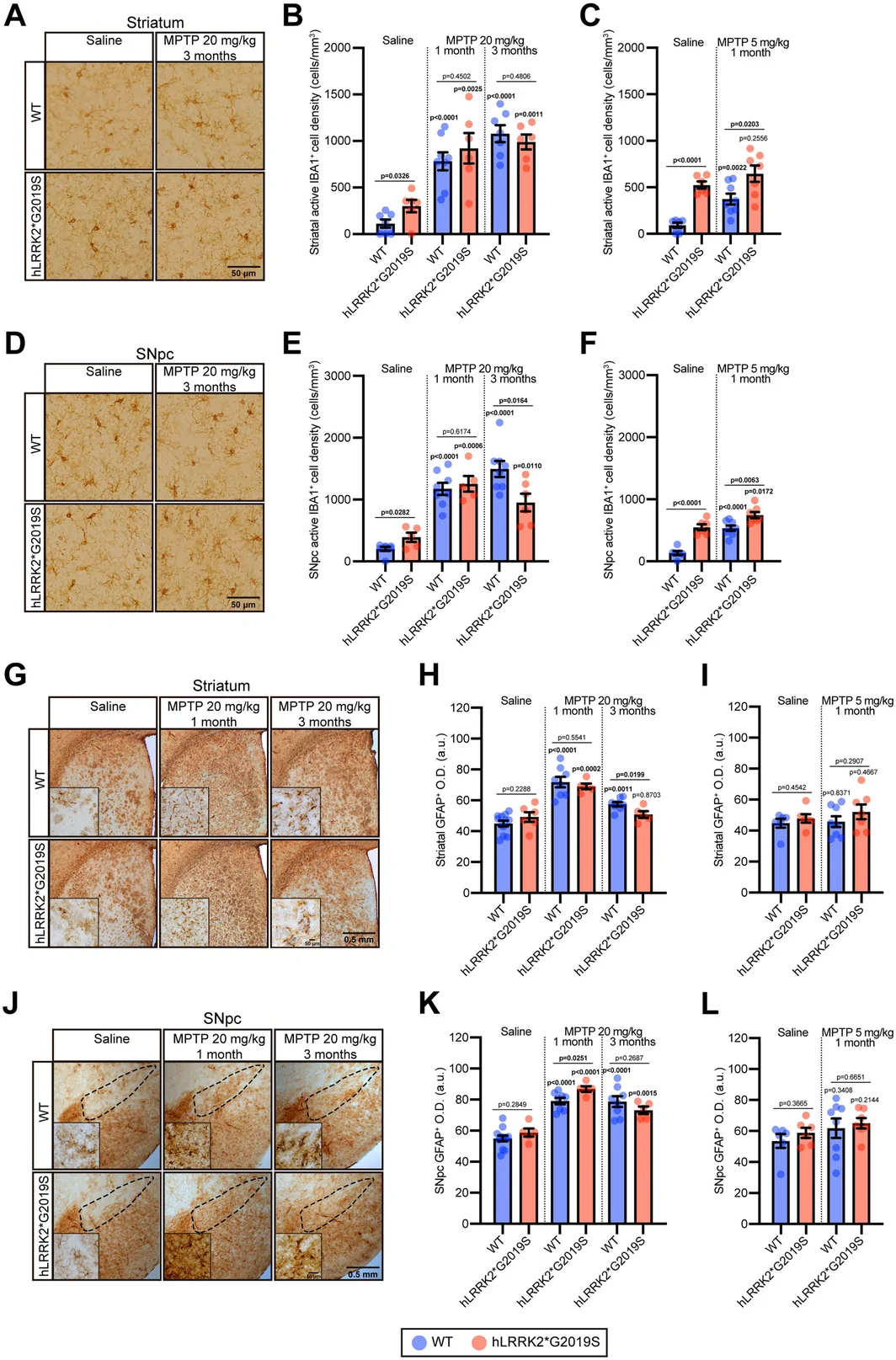
Nigrostriatal glial response of WT and hLRRK2*G2019S mice following MPTP‐induced parkinsonism. (A–F) Nigrostriatal microglial activation of WT and hLRRK2*G2019S mice subjected to the standard chronic MPTP treatment, 20 mg/kg for 1 or 3 months, or the sub‐threshold MPTP treatment, 5 mg/kg for 1 month, displaying high magnification images of Iba1^+^ cells from mice treated with MPTP 20 mg/kg for 3 months (A, D) together with the stereological quantification of active microglia for both MPTP protocols in the striatum (B, C) and SNpc (E, F). (G–L) Astroglial response in the nigrostriatal pathway, of previously described experimental groups, showing representative images of coronal sections after GFAP immunostaining and the corresponding analysis of GFAP^+^ O.D. in the striatum (G–I) and SNpc (J–L). Insets in panels G and J show representative high magnification images of GFAP^+^ staining in the striatum and SNpc from the different experimental groups. Dashed lines in panel G delineate the SNpc. *p*‐values above connecting lines indicate pairwise statistical comparisons. Individual *p*‐values above each bar indicate comparisons with the saline controls of the corresponding genotype.

The glial response occurring in the nigrostriatal pathway of hLRRK2*G2019S and WT mice subjected to chronic MPTP intoxication was studied, either with the standard dose (20 mg/kg, for 1 and 3 months) or the sub‐threshold dose (5 mg/kg for 1 month). Microglial response in the nigrostriatal pathway was assessed by stereological quantification of Iba1^+^ cell morphology as indicated before. In saline treated mice, hLRRK2*G2019S mice displayed a significant higher density of active microglia, both in striatum and SNpc (Figure 7A–F), as described before for non‐treated young adults of this genotype (Figure 5A–D). The standard MPTP treatment, 20 mg/kg, induced in WT mice a progressive reactive microglial response, producing an important increase in the density of nigrostriatal active microglia at the end of the neurotoxic treatment (Figure 7A,B,D,E), as previously described (Muñoz‐Manchado et al. 2016; Villadiego, Labrador‐Garrido, et al. 2018; Villadiego, Romo‐Madero, et al. 2018). After 1 month of MPTP administration, hLRRK2*G2019S mice showed similar levels of active microglial density, both in striatum and SNpc, than WT saline controls. However, at the end of the neurotoxic treatment, hLRRK2*G2019S mice presented lower microgliosis in the SNpc than their WT counterparts (Figure 7E). The sub‐threshold MPTP treatment, 5 mg/kg, also increased reactive microglial density in both genotypes (Figure 7C,F), indicating that although the sub‐threshold MPTP dosage did not induce detectable dopaminergic damage, it elicited a significant microglial response. Because hLRRK2*G2019S mice exhibited higher basal microgliosis, genotype‐specific microglial responses to chronic MPTP administration were further evaluated by calculating the fold change in active microglial density relative to the corresponding genotype‐matched saline‐treated group. This analysis revealed a significant lower microglial response to the chronic MPTP treatment in hLRRK2*G2019S mice than in WT mice (Figure S4A–D).

Astrocytic response was assessed through GFAP^+^ immunoreactivity measurements as previously stated. No differences in astrogliosis were observed between saline‐treated WT and hLRRK2*G2019S mice in either of the two MPTP protocols (Figure 7G–L). In the striatum of mice subjected to the standard neurotoxic treatment (20 mg/kg), after 1 month of MPTP administration, a sharp increase in GFAP^+^ O.D. was observed in animals of both genotypes, without significant differences between them (Figure 7G,H). At the 3‐month endpoint, striatal GFAP^+^ O.D. decreased with respect to the 1‐month treatment point, but it still maintained higher levels than the animals treated with saline. However striatal GFAP^+^ immunostaining was significantly lower in hLRRK2*G2019S mice than in WT mice (Figure 7G,H). In the SNpc, after 1 month of the standard MPTP treatment, a similar increase in GFAP^+^ O.D. was detected in both genotypes (Figure 7J,K). At the 3‐month timepoint, high levels of astroglial reaction were maintained in WT MPTP‐treated mice, while the hLRRK2*G2019S MPTP‐treated group showed a trend toward reducing astrogliosis, being lower than the observed at the 1‐month MPTP treatment timepoint on this experimental group (Figure 7J,K). In contrast, the sub‐threshold dose of MPTP (5 mg/kg) did not induce detectable astrogliosis in either the striatum or SNpc of WT or hLRRK2G2019S mice (Figure 7I,L). Genotype‐specific astroglial responses were also evaluated by calculating the fold change in GFAP immunoreactivity relative to the corresponding genotype‐matched saline‐treated group. This analysis showed comparable astroglial responses in WT and hLRRK2G2019S mice after 1 month of either the standard or sub‐threshold MPTP regimen. In contrast, following 3 months of the standard chronic MPTP treatment, hLRRK2G2019S mice exhibited a significantly lower astroglial response than WT mice (Figure S4E).

**FIGURE 8:**
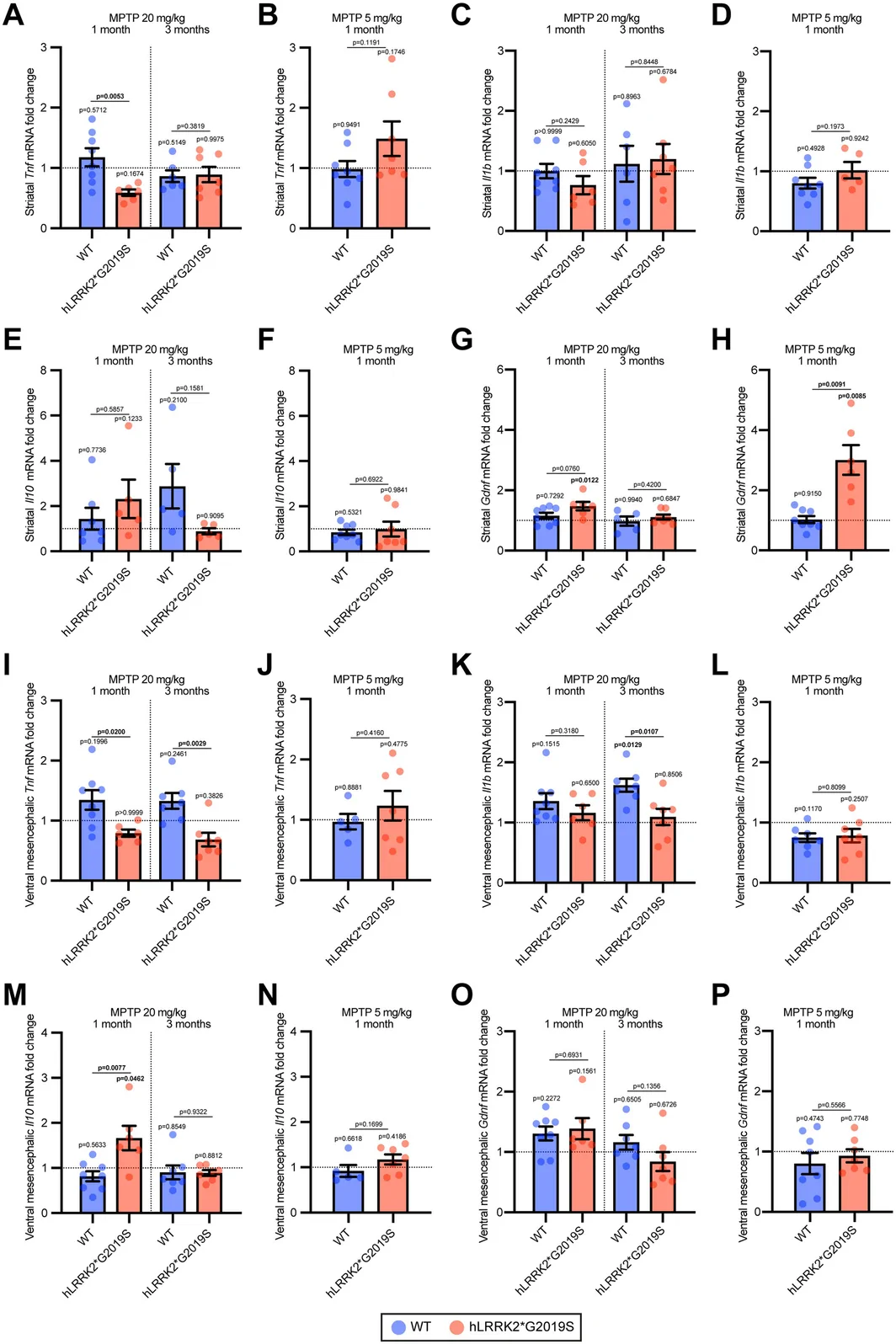
Chronic MPTP‐induced changes in mRNA expression of inflammatory cytokines and *Gdnf* in WT and hLRRK2*G2019S mice. (A–H) Fold change in striatal mRNA expression of *Tnf* (A, B), *Il1b* (C, D), *Il10* (E, F) and *Gdnf* (G, H) of WT and hLRRK2*G2019S mice following the standard chronic (20 mg/kg for 1 or 3 months) or sub‐threshold (5 mg/kg for 1 month) MPTP treatments. (I–P) Fold change in ventral mesencephalic mRNA expression of *Tnf* (I, J), *Il1b* (K, L), *Il10* (M, N) and *Gdnf* (0, P) in the previously described experimental groups. The dashed horizontal line at y = 1 represents the mean mRNA expression level of the corresponding genotype‐matched saline group. *p*‐values above connecting lines indicate pairwise statistical comparisons. Individual *p*‐values above each bar indicate comparisons with the corresponding genotype‐matched saline group.

To further characterize the neuroglial response associated with chronic MPTP treatment, we analyzed treatment‐induced changes in the mRNA expression of the previously examined cytokines (*Il1b, Il6, Tgfb1, Tnf, Ifng, Il10*, and *Il4*) and neurotrophic factors (*Gdnf* and *Bdnf*). Following the standard MPTP protocol, hLRRK2G2019S mice exhibited lower striatal *Tnf* mRNA levels than WT mice after 1 month of treatment (Figure 8A). Likewise, *Tnf* mRNA expression in the ventral mesencephalon was lower in hLRRK2G2019S than in WT mice after both 1 and 3 months of the standard MPTP dosage (Figure 8I), whereas *Il1b* mRNA expression was reduced only after 3 months of treatment (Figure 8K). In addition, striatal mRNA levels of the immunosuppressive cytokine *Il10* were higher in hLRRK2G2019S than in WT mice after 1 month of this treatment (Figure 8M). Also, *Gdnf* mRNA expression in the striatum (Figure 8G) and *Bdnf* mRNA expression in the ventral mesencephalon (Table S1) showed a transient trend toward increased expression after 1 month of the standard MPTP treatment in hLRRK2*G2019S mice. In contrast, no treatment‐related changes were detected in the expression of *Il6, Ifng, Tgfb1* or *Il4* in the striatum or ventral mesencephalon of either genotype (Table S1). The sub‐threshold MPTP treatment increased *Gdnf* mRNA expression in the striatum (Figure 8H) and *Il4* mRNA expression in the ventral mesencephalon of hLRRK2G2019S mice (Table S1). No other significant changes were detected in the mRNA expression of the cytokines or neurotrophic factors analyzed in either the striatum or ventral mesencephalon of WT or hLRRK2G2019S mice (Figure 8 and Table S1). Overall, both the histological and the mRNA expression analyzes suggest that the hLRRK2 G2019S mutation mitigates the long‐term neuroglial response associated with chronic MPTP‐induced neurodegeneration.

## 4 Discussion

Despite the high frequency of LRRK2 G2019S mutation in PD patients, the relevant role in PD pathophysiology, and the different animal models carrying this mutation reported (Taymans et al. 2023; Seegobin et al. 2020), its specific contribution to nigrostriatal neurodegeneration still remains elusive. In this study, we performed a comprehensive histological, neurochemical and behavioral analysis of a mouse model expressing the human LRRK2 G2019S mutation, both in young adults (3–6 months old) or aged (16–18 months old) animals. Consistent with the low penetrance of this mutation (Lee et al. 2017) and with other rodent models described (Garcia‐Miralles et al. 2015; Seegobin et al. 2020), we did not detect evidence of dopaminergic nigrostriatal degeneration neither in young adults nor aged mice. Nevertheless, despite the lack of nigrostriatal neurodegeneration, hLRRK2*G2019S mice showed deficits in gait coordination in the early adulthood, and a clear hyperactive phenotype during the aged stage. The hyperactive phenotype observed in aged hLRRK2*G2019S mice contrasts with the bradykinesia typically expected in experimental models of PD and with the age‐related decline in motor performance. However, previous studies using hLRRK2G2019S knock‐in mice also reported a marked age‐dependent hyperactive phenotype in the absence of nigrostriatal neurodegeneration. This behavioral alteration was attributed to the enhanced kinase activity conferred by the G2019S mutation, as both kinase‐dead LRRK2 (D1994S) transgenic mice and pharmacological inhibition of LRRK2 kinase activity reversed the age‐associated hyperactivity (Longo et al. 2014). In addition, recent works using the hLRRK2*G2019S transgenic line employed here also described a hyperactive phenotype in 10–12 months old mice without evidence of nigrostriatal dopaminergic degeneration (Skiteva et al. 2022; Yao et al. 2022). Interestingly, hLRRK2*G2019S transgenic mice exhibit alterations in midbrain glutamatergic synapses (Sitzia et al. 2022; Skiteva et al. 2022), suggesting that the hyperactive phenotype may arise from changes in neuronal neurotransmission. Moreover, we identified a significant overexpression of hLRRK2*G2019S in striatal cholinergic interneurons, a neuronal population that critically regulates corticostriatal neurotransmission and motor behavior (Tanimura et al. 2018). Although exploring the functional consequences of the LRRK2 G2019S mutation in these cholinergic interneurons falls beyond the scope of this study, future research should focus on elucidating its impact within this specific neuronal population and its relationship with motor alterations.

Mitochondrial dysfunction has been strongly related with the pathophysiology of idiopathic and genetic PD (Henrich et al. 2023). Different in vitro studies, using fibroblast, cortical neurons and induced pluripotent stem cell‐derived dopaminergic neurons, have reported mitochondrial alterations associated to LRRK2 PD mutations (Oun et al. 2022; Korecka et al. 2019; Williamson et al. 2023; Valderhaug et al. 2024). Moreover, abnormal mitochondrial morphology has been shown in aged LRRK2 G2019S knock‐in mice (Yue et al. 2015). In the present study, we studied in vivo how the human LRRK2 G2019S mutation would affect the susceptibility of rodent dopaminergic SNpc neurons to chronic mitochondrial inhibition. To address that we used two complementary MPTP administration regimens. First, we employed a previously validated model of long‐term administration of the selective dopaminergic mitochondrial complex I inhibitor (20 mg/kg for 1 or 3 months), that resembles the slow and progressive neurodegenerative process characteristic of the disease (Muñoz‐Manchado et al. 2016; Villadiego, Romo‐Madero, et al. 2018; Villadiego, Muñoz‐Manchado, et al. 2023). Second, we used a sub‐threshold MPTP protocol (5 mg/kg, for 1 month), designed to induce minimal or no dopaminergic damage. Remarkably, neither chronic MPTP protocols increased dopaminergic nigrostriatal neurodegeneration or exacerbated motor alterations in hLRRK2*G2019S transgenic mice, suggesting that this mutation does not potentiate in vivo the dopaminergic damage induced by chronic mitochondrial inhibition. These findings differ from previous reports showing increased susceptibility to MPTP‐induced nigrostriatal degeneration in transgenic or knock‐in hLRRK2G2019S mice (Karuppagounder et al. 2016; Arbez et al. 2020; Novello et al. 2022). However, an important methodological difference between those studies and the present work is the MPTP administration regimen employed. Previous studies used acute or subacute MPTP regimens (1–7 days of neurotoxin administration) and evaluated dopaminergic degeneration within 7–10 days after treatment initiation, whereas the present study was based on a chronic MPTP treatment (28–84 days), with nigrostriatal degeneration assessed 38–98 days after treatment onset. Acute and subacute MPTP protocols are characterized by their reversibility, producing a transient reduction in TH expression during the early stages of neurotoxin exposure that, in many cases, is not accompanied by dopaminergic neuronal death (Eidelberg et al. 1986; Petroske et al. 2001). Therefore, the differences between the present study and previous reports may reflect the fact that these different MPTP paradigms assess distinct phases of the neurotoxic process rather than conflicting biological effects. Thus, it is conceivable that transgenic or knock‐in hLRRK2G2019S mice are more susceptible to the transient downregulation of TH occurring during the initial days of MPTP exposure. By contrast, the chronic MPTP model used here (20 mg/kg for 1 or 3 months), which produces stable and irreversible degeneration of the nigrostriatal pathway (Muñoz‐Manchado et al. 2016), resulted in comparable dopaminergic neurodegeneration in hLRRK2*G2019S and WT mice. Moreover, recent works exploring R1441C and G2019S mutations in mitochondrial alterations indicated a more accused mitochondrial dysfunction in neuronal cells with R1441C mutation (Williamson et al. 2023) and prevalent in vivo effects of G2019S on microglial cells without alterations in SNpc dopaminergic neurons (Singh et al. 2021), suggesting divergent cell‐type specificity and mechanism of action of the different LRRK2 mutations on the mitochondrial function.

Emerging evidences points to a prominent role of the neuroimmune response in PD pathogenesis and prognosis (Roodveldt et al. 2024). In this way, different studies have highlighted the important role of LRRK2 in peripheral immunity, however its specific contribution to glial response associated to neurodegeneration is still poorly understood (Russo et al. 2022; Tsafaras and Baekelandt 2022; Cabezudo et al. 2023). Our morphological analysis show that the expression of hLRRK2 G2019S results in an accelerated microglial activation during the young adult stage, anticipating the characteristic aged‐related microgliosis (Sheng et al. 1998; Wong 2013). In addition, changes in mRNA expression of cytokines as *Il1b, Il6*, and *Tnf* was founded in the nigrostriatal pathway of hLRRK2*G2019S mice. Supporting these findings, a recent study demonstrated an increased proportion of disease‐associated microglia in hLRRK2 G2019S Knock‐in mice, also in the context of absence of dopaminergic loss, suggesting a relevant microglial response in prodromal stages of the disease (Iovino et al. 2024). Our work also addresses the study of the glial response related to MPTP‐induced nigrostriatal degeneration. Remarkably, in contrast with the microglial activation observed without dopaminergic damage, our data indicates a reduction of glial activation, both in astroglia and microglia, in hLRRK2*G2019S mice after dopaminergic neurodegeneration (3 months of MPTP treatment). This was accompanied by reduced mRNA expression of the pro‐inflammatory cytokines *Tnf* and *Il1b*, along with increased expression levels of the immunosuppressive cytokine *Il10*. Taken together, our findings suggest a differential modulation of the neuroinflammatory response depending on the context of dopaminergic damage. Future studies should explore the cellular and molecular mechanism underlying LRRK2‐mediated neuroimmune response both in prodromal and advances stages of the disease.

## Author Contributions

**Ernesto García‐Roldán:** methodology, formal analysis, data curation. **Juan José Toledo‐Aral:** conceptualization, methodology, writing – original draft, writing – review and editing, supervision, resources, project administration, funding acquisition. **Laura Morón‐Márquez:** methodology, formal analysis, data curation. **Roberto García‐Swinburn:** conceptualization, methodology, formal analysis, data curation, visualization, writing – review and editing. **Javier Villadiego:** conceptualization, methodology, visualization, writing – original draft, writing – review and editing, supervision, project administration, funding acquisition. **Reposo Ramírez‐Lorca:** methodology, formal analysis, data curation. **Carmen Conde‐Naranjo:** methodology, formal analysis, data curation. **Alfonso Rodríguez‐Gil:** methodology, formal analysis, data curation. **Isabel Espadas:** methodology, formal analysis, data curation.

## Funding

This research was supported by the Spanish Ministries of Economy and Competitiveness, and Science and Innovation/Spanish Research Agency/10.13039/501100011033 grant nos. RTC‐2015‐3309‐1 (J.J.T.‐A.); PID2019‐105995RB‐I00 (J.J.T.‐A. and J.V.); PID2023‐146386NB‐I00 (J.J.T.‐A. and J.V.). Moreover, this research was also funded by Red TerCel ISCIII (no. RD16/0011/0025 to J.J.T.‐A.); Consejería de Economía, Conocimiento, Empresas y Universidad US‐1380891 (to J.J.T.‐A. and J.V.) and P20_01130 (J.V.); and Consejería de Salud y Familias, Junta de Andalucía grant no. PECOVID‐0078‐2020 (J.V.). The funders had no role in study design, data collection and analysis, decision to publish or preparation of the manuscript.

## Conflicts of Interest

The authors declare no conflicts of interest.

## Supporting information


**Figure S1:** mRNA expression levels of Tnf, Ifng, Il10, Il4, and Bdnf in young and aged WT and hLRRK2*G2019S mice. (A–J) Relative mRNA expression of Tnf (A, F), Infg (B, G), Il10 (C, H), Il4 (D, I) and Bdnf (E, J) in the striatum and ventral mesencephalon of young adult and aged WT and hLRRK2*G2019S mice. *p*‐values above connecting lines indicate pairwise statistical comparisons. Individual *p*‐values above each bar indicate comparisons with the young controls of the corresponding genotype.
**Figure S2:** Openfield analysis of WT and hLRRK2*G2019S mice subjected to chronic MPTP treatment. (A) Openfield tracking of WT and hLRRK2*G2019S mice treated with saline or MPTP (20 mg/kg for 3 months). (B–Q) Openfield locomotor parameters of WT and hLRRK2*G2019S mice chronically treated with saline or MPTP (20 mg/kg for 3 months or 5 mg/kg for 1 month) showing total track length (B, C); activity (D, E); average speed (F, G); percentage of time spent at low (< 20m/s; H, I), intermediate (20–60m/s; J, K), and high (> 60m/s; L, M) velocities; and vertical activity indicating the rearings counts (N, O) and rearings duration (P, Q). *p*‐values above connecting lines indicate pairwise statistical comparisons. Individual *p*‐values above each bar indicate comparisons with the saline‐treated controls of the corresponding genotype.
**Figure S3:** Footprint analysis of WT and hLRRK2*G2019S mice chronically treated with MPTP. (A‐H) Footprint analysis from saline and MPTP‐treated WT and hLRRK2*G2019S mice, receiving either 20 mg/kg (1 or 3 months) or 5 mg/kg (1 month) of MPTP. Forelimb stride length (A, B) and variance (C, D) and hindlimbs stride length (E, F) and variance (G, H) are shown. *p*‐values above connecting lines indicate pairwise statistical comparisons. Individual *p*‐values above each bar indicate comparisons with the saline‐treated controls of the corresponding genotype.
**Figure S4:** Microglial and astroglial responses in the nigrostriatal pathway following chronic MPTP treatment. (A–D) Fold change in the density of active microglia in the striatum (A, B) and SNpc (C, D) of WT and hLRRK2*G2019S mice treated with the standard MPTP (20 mg/kg for 1 or 3 months) or the sub threshold (5 mg/kg for 1 month) treatments. (E, F) Fold change in the astroglial GFAP^+ ^O.D. in the striatum (E, F) and the SNpc (G, H) of the previously described experimental groups. Fold change was calculated by normalizing each MPTP‐treated group to the mean value of its corresponding genotype‐matched saline treated group. *p*‐values above connecting lines indicate pairwise statistical comparisons.
**Table S1:** Chronic MPTP‐induced changes in mRNA expression of Il6, Ifng, Tgfb1, Il4 and Bdnf in WT and hLRRK2*G2019S mice. Fold change in striatal and ventral mesencephalic mRNA expression of Il6, Ifng, Tgfb1, Il4 and Bdnf of WT and hLRRK2*G2019S mice following the standard chronic (20 mg/kg for 1 or 3 months) or sub‐threshold (5 mg/kg for 1 month) MPTP treatments. Data are presented as mean ± SEM. *p*‐values indicate pairwise statistical comparisons. *p*‐values in superscript indicate comparisons with the corresponding genotype matched saline group. Statistical report. Statistical data corresponding to Figures 3, 4, 5, 6, 7, 8 and Figures S1–S5.

## Data Availability

The data that support the findings of this study are openly available upon request to the corresponding authors and in Supporting Information.
