# Supplementary material for: Neuroinflammatory and Motor Alterations in LRRK2*G2019S Transgenic Mice Without Enhanced Vulnerability to Aging or Chronic MPTP‐Induced Nigrostriatal Neurodegeneration
Source: J Neurochem. 2026 Sep 9;170(9):e70547. doi: 10.1111/jnc.70547 (PMC13555517; doi:10.1111/jnc.70547)
Supplement: Supplementary file 1 [file JNC-170-e70547-s001.pdf]

Supplementary Figure 1

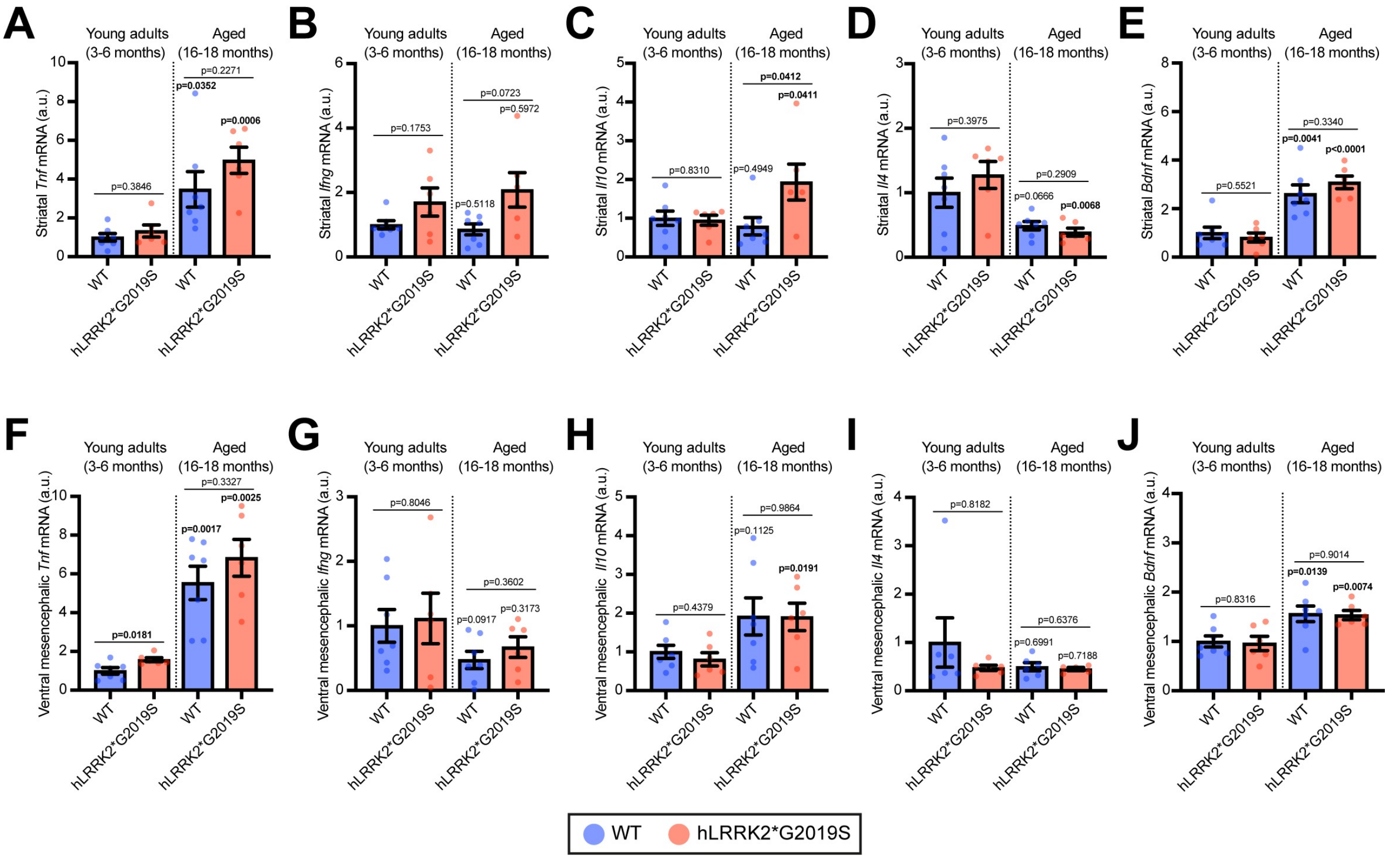

Supplementary Figure 1. mRNA expression levels of *Tnf, Ifng, Il10, Il4* and *Bdnf* in young and aged WT and hLRRK2*G2019S mice. (A-J) Relative mRNA expression of *Tnf* (A,F), *Infg* (B,G), *Il10* (C,H), *Il4* (D,I) and *Bdnf* (E,J) in the striatum and ventral mesencephalon of young adult and aged WT and hLRRK2*G2019S mice. p-values above connecting lines indicate pairwise statistical comparisons. Individual p-values above each bar indicate comparisons with the young controls of the corresponding genotype.

Supplementary Figure 2

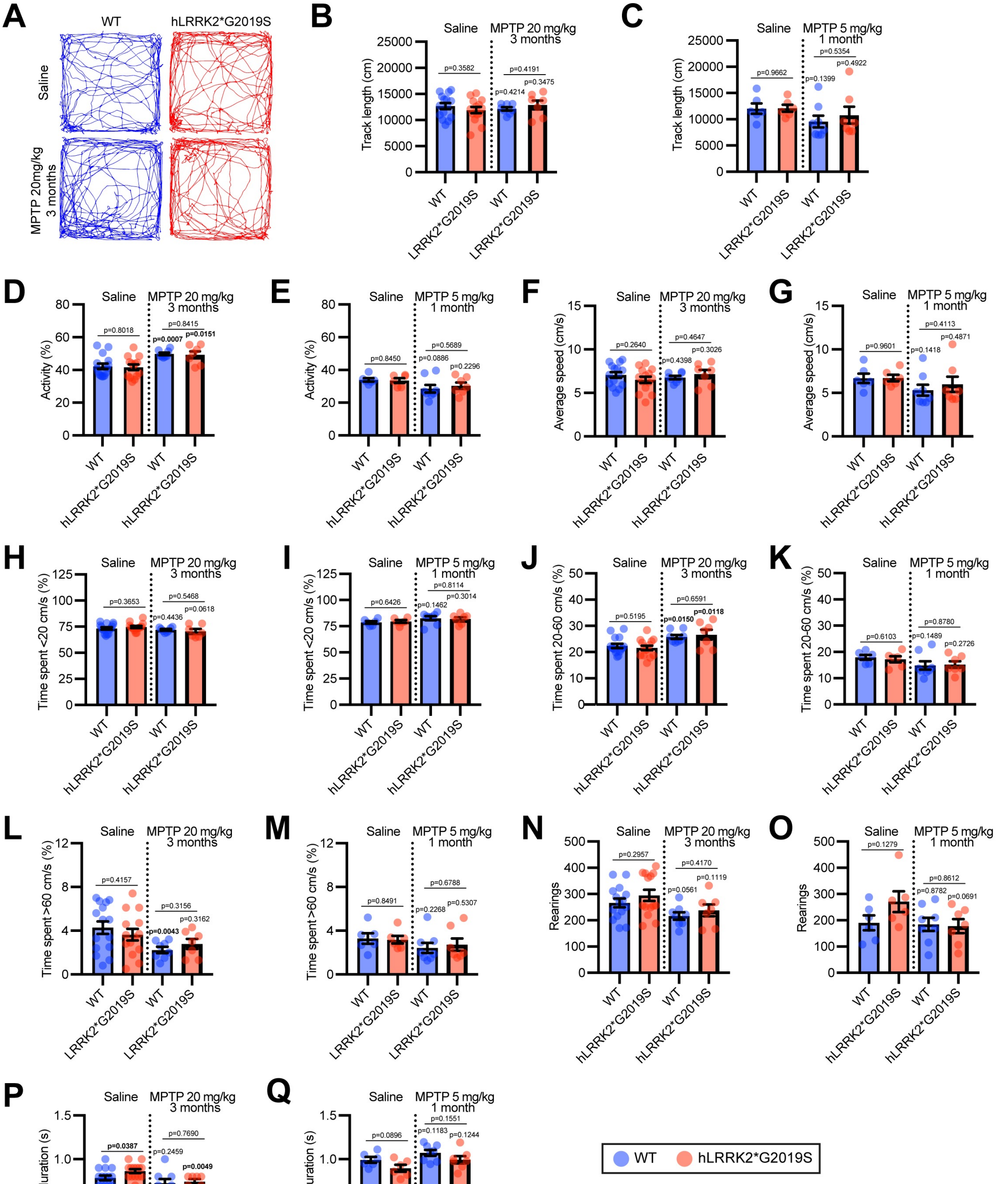

Supplementary Figure 2. Openfield analysis of WT and hLRRK2*G2019S mice subjected to chronic MPTP treatment. (A) Openfield tracking of WT and hLRRK2*G2019S mice treated with saline or MPTP (20mg/kg for 3 months). (B-Q) Openfield locomotor parameters of WT and hLRRK2*G2019S mice chronically treated with saline or MPTP (20 mg/kg for 3 months or 5 mg/kg for 1 month) showing total track length (B,C); activity (D,E); average speed (F,G); percentage of time spent at low (<20m/s; H,I), intermediate (20-60m/s; J,K), and high (>60m/s; L,M) velocities; and vertical activity indicating the rearings counts (N,O) and rearings duration (P,Q). p-values above connecting lines indicate pairwise statistical comparisons. Individual p-values above each bar indicate comparisons with the saline-treated controls of the corresponding genotype.

Supplementary Figure 3

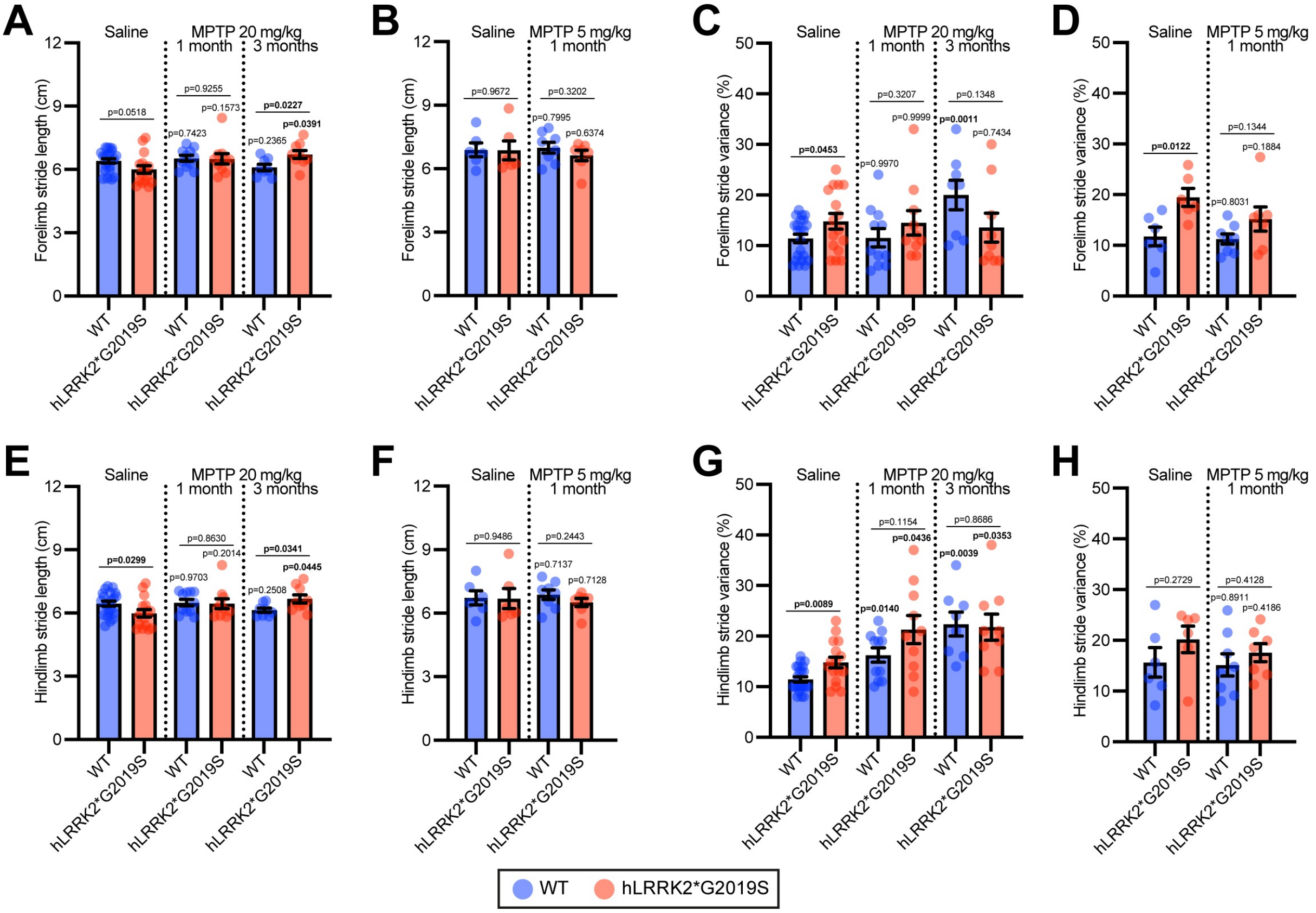

Supplementary Figure 3. Footprint analysis of WT and hLRRK2*G2019S mice chronically treated with MPTP. (A-H) Footprint analysis from saline and MPTP-treated WT and hLRRK2*G2019S mice, receiving either 20 mg/kg (1 or 3 months) or 5 mg/kg (1 month) of MPTP. Forelimb stride length (A,B) and variance (C,D) and hindlimbs stride length (E,F) and variance (G,H) are shown. p-values above connecting lines indicate pairwise statistical comparisons. Individual p-values above each bar indicate comparisons with the saline-treated controls of the corresponding genotype.

Supplementary Figure 4

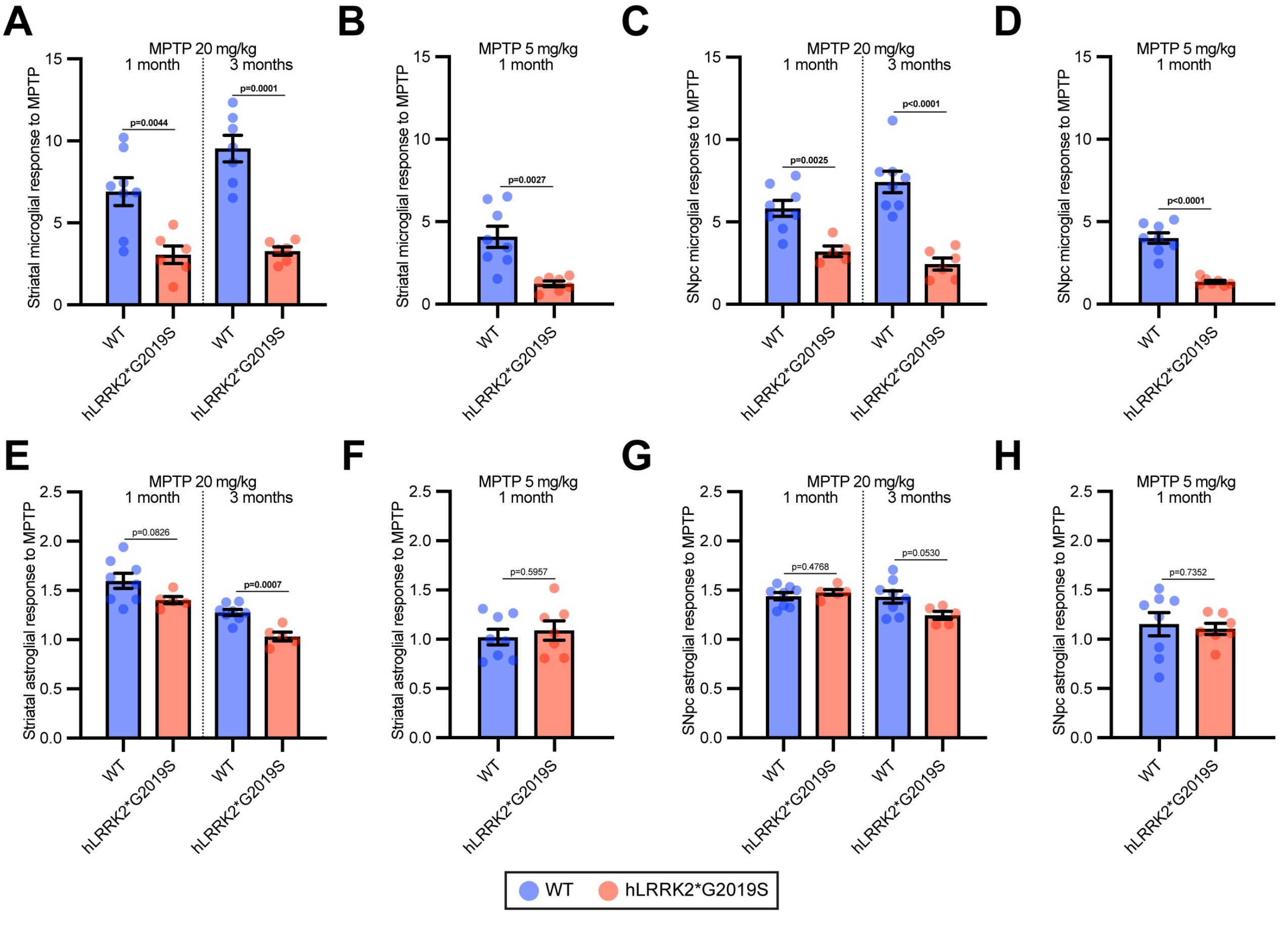

Supplementary Figure 4. Microglial and astroglial responses in the nigrostriatal pathway following chronic MPTP treatment. (A-D) Fold change in the density of active microglia in the striatum (A,B) and SNpc (C,D) of WT and hLRRK2*G2019S mice treated with the standard MPTP (20 mg/kg for 1 or 3 months) or the sub-threshold (5 mg/kg for 1 month) treatments. (E-F) Fold change in the astroglial GFAP^{+} O.D. in the striatum (E,F) and the SNpc (G,H) of the previously described experimental groups. Fold change was calculated by normalizing each MPTP-treated group to the mean value of its corresponding genotype-matched saline-treated group. p-values above connecting lines indicate pairwise statistical comparisons.

Supplementary Table 1

		Standard chronic MPTP treatment						Sub-threshold chronic MPTP treatment		
		MPTP 20 mg/kg, 1 month			MPTP 20 mg/kg, 3 months			MPTP 5 mg/kg, 1 month		
	**Gene**	**WT**	**hLRRK2*G2019S**	**p value**	**WT**	**hLRRK2*G2019S**	**p value**	**WT**	**hLRRK2*G2019S**	**p value**
**Striatum**	*Il6*	0.941 ± 0.0672 $^{p=0.8414}$	1.111 ± 0.0883 $^{p=0.6302}$	p=0.1447	0.7633 ± 0.0602 $^{p=0.1461}$	0.7914 ± 0.0752 $^{p=0.2066}$	p=0.8357	0.8026 ± 0.0613 $^{p=0.1036}$	0.8196 ± 0.0983 $^{p=0.1625}$	p=0.8826
	Ifng	1.565 ± 0.1645 $^{p=0.1243}$	1.206 ± 0.2035 $^{p=0.4274}$	p=0.1910	1.235 ± 0.3266 $^{p=0.7086}$	0.8131 ± 0.1596 $^{p>0.9999}$	p=0.2322	1.510 ± 0.3271 $^{p=0.2345}$	1.144 ± 0.1690 $^{p=0.5752}$	p=0.3877
	Tgfb1	1.102 ± 0.0637 $^{p=0.7202}$	1.075 ± 0.0341 $^{p=0.8108}$	p=0.7390	0.7789 ± 0.1045 $^{p=0.3025}$	0.8595 ± 0.0699 $^{p=0.4697}$	p=0.5238	1.185 ± 0.0573 $^{p=0.1720}$	1.088 ± 0.0663 $^{p=0.3191}$	p=0.2846
	Il4	1.521 ± 0.6619 $^{p=0.6476}$	1.583 ± 0.7553 $^{p>0.9999}$	p>0.9999	0.556 ± 0.1890 $^{p=0.7764}$	1.130 ± 0.4540 $^{p>0.9999}$	p=0.3351	1.084 ± 0.3569 $^{p=0.8624}$	0.984 ± 0.1521 $^{p=0.9513}$	p=0.6126
	Bdnf	1.295 ± 0.1416 $^{p=0.2629}$	1.286 ± 0.0965 $^{p=0.0677}$	p=0.9614	0.820 ± 0.1382 $^{p=0.6196}$	1.004 ± 0.0517 $^{p=0.9991}$	p=0.2562	1.270 ± 0.0812 $^{p=0.0617}$	1.153 ± 0.0832 $^{p=0.2012}$	p=0.3367
**Ventral mesencephalon**	*Il6*	1.055 ± 0.0718 $^{p=0.7876}$	1.190 ± 0.0776 $^{p=0.1711}$	p=0.2307	0.9267 ± 0.0634 $^{p=0.6759}$	0.7547 ± 0.0606 $^{p=0.0545}$	p=0.0735	0.8394 ± 0.0419 $^{p=0.2046}$	1.048 ± 0.0868 $^{p=0.6885}$	p=0.0516
	Ifng	1.031 ± 0.2009 $^{p=0.9885}$	0.899 ± 0.3132 $^{p=0.9343}$	p=0.7195	0.828 ± 0.1065 $^{p=0.7250}$	1.010 ± 0.1766 $^{p=0.9993}$	p=0.4557	1.123 ± 0.2233 $^{p=0.7442}$	1.149 ± 0.1626 $^{p=0.6703}$	p=0.9275
	Tgfb1	1.218 ± 0.0584 $^{p=0.1138}$	1.205 ± 0.0752 $^{p=0.3529}$	p=0.8923	1.140 ± 0.1498 $^{p=0.4523}$	1.045 ± 0.0841 $^{p=0.9406}$	p=0.5648	0.9477 ± 0.0246 $^{p=0.3768}$	0.9713 ± 0.0266 $^{p=0.4791}$	p=0.5263
	Il4	1.140 ± 0.1405 $^{p=0.5419}$	1.463 ± 0.6155 $^{p>0.9999}$	p=0.6943	0.983 ± 0.1257 $^{p=0.9841}$	1.172 ± 0.1803 $^{p=0.8204}$	p=0.4070	0.566 ± 0.0604 $^{p=0.0536}$	0.771 ± 0.0701 $^{p=0.1561}$	**p=0.0445**
	Bdnf	1.442 ± 0.1047 $^{\mathbf{p=0.0345}}$	1.293 ± 0.1339 $^{p=0.3573}$	p=0.3881	1.351 ± 0.2081 $^{p=0.1572}$	0.968 ± 0.1249 $^{p=0.9848}$	p=0.1248	0.967 ± 0.0428 $^{p=0.6206}$	0.987 ± 0.0467 $^{p=0.8608}$	p=0.7591

Supplementary Table 1. Chronic MPTP-induced changes in mRNA expression of *Il6, Ifng, Tgfb1, Il4* and *Bdnf* in WT and hLRRK2*G2019S mice. Fold change in striatal and ventral mesencephalic mRNA expression of *Il6, Ifng, Tgfb1, Il4* and *Bdnf* of WT and hLRRK2*G2019S mice following the standard chronic (20 mg/kg for 1 or 3 months) or sub-threshold (5 mg/kg for 1 month) MPTP treatments. Data are presented as mean ± SEM. p-values indicate pairwise statistical comparisons. p-values in superscript indicate comparisons with the corresponding genotype-matched saline group.

Statistical report. Statistical data corresponding to Figures 3-8 and Supplementary Figures 1-5.

Statistical report: Figure 3.

Figure 3				
**Figure**	**Experimental groups compared**	**Test statistic**	**P value**	**Statistical test**
3B	Young adults: WT vs hLRRK2*G2019S	t=0.5380	p=0.5962	Unpaired two-tailed t-test with Welch's correction
	Aged: WT vs hLRRK2*G2019S	t=0.5619	p=0.5831	Unpaired two-tailed t-test
	WT: Young adults vs Aged	t=3.764	p=0.0011	Unpaired two-tailed t-test
	hLRRK2*G2019S: Young adults vs Aged	t=2.364	p=0.0440	Unpaired two-tailed t-test with Welch's correction
3D	Young adults: WT vs hLRRK2*G2019S	t=0.5543	p=0.5828	Unpaired two-tailed t-test
	Aged: WT vs hLRRK2*G2019S	t=0.3648	p=0.7180	Unpaired two-tailed t-test
	WT: Young adults vs Aged	t=5.514	p<0.0001	Unpaired two-tailed t-test
	hLRRK2*G2019S: Young adults vs Aged	t=5.873	p<0.0001	Unpaired two-tailed t-test with Welch's correction
3E	Young adults: WT vs hLRRK2*G2019S	U=72730	p=0.8346	U-Mann Whitney test
	Aged: WT vs hLRRK2*G2019S	U=53672	p=0.0171	U-Mann Whitney test
	WT: Young adults vs Aged	U=64509	p=0.1222	U-Mann Whitney test
	hLRRK2*G2019S: Young adults vs Aged	U=61514	p=0.4266	U-Mann Whitney test
3F	Young adults: WT vs hLRRK2*G2019S	t=0.2357	p=0.8162	Unpaired two-tailed t-test with Welch's correction
	Aged: WT vs hLRRK2*G2019S	t=1.093	p=0.2907	Unpaired two-tailed t-test
	WT: Young adults vs Aged	t=5.806	p<0.0001	Unpaired two-tailed t-test
	hLRRK2*G2019S: Young adults vs Aged	t=1.920	p=0.0673	Unpaired two-tailed t-test
3G	Young adults: WT vs hLRRK2*G2019S	t=1.040	p=0.340	Unpaired two-tailed t-test
	Aged: WT vs hLRRK2*G2019S	t=0.5446	p=0.5935	Unpaired two-tailed t-test
	WT: Young adults vs Aged	U=127	p=0.9194	U-Mann Whitney test
	hLRRK2*G2019S: Young adults vs Aged	t=0.8951	p=0.3800	Unpaired two-tailed t-test
3H	Young adults: WT vs hLRRK2*G2019S	t=0.6451	p=0.5223	Unpaired two-tailed t-test
	Aged: WT vs hLRRK2*G2019S	t=0.9861	p=0.3387	Unpaired two-tailed t-test
	WT: Young adults vs Aged	t=1.104	p=0.2769	Unpaired two-tailed t-test
	hLRRK2*G2019S: Young adults vs Aged	U=68	p=0.8460	U-Mann Whitney test
3I	Young adults: WT vs hLRRK2*G2019S	t=0.4483	p=0.6562	Unpaired two-tailed t-test
	Aged: WT vs hLRRK2*G2019S	t=0.04089	p=0.9679	Unpaired two-tailed t-test
	WT: Young adults vs Aged	t=2.785	p=0.0203	Unpaired two-tailed t-test with Welch's correction
	hLRRK2*G2019S: Young adults vs Aged	t=3.461	p=0.0022	Unpaired two-tailed t-test

Statistical report: Figure 4.

Figure 4				
**Figure**	**Experimental groups compared**	**Test statistic**	**P value**	**Statistical test**
4B	Young adults: WT vs hLRRK2*G2019S	t=0.4692	p=0.6416	Unpaired two-tailed t-test
	Aged: WT vs hLRRK2*G2019S	t=3.505	p=0.0017	Unpaired two-tailed t-test
	WT: Young adults vs Aged	t=1.686	p=0.1010	Unpaired two-tailed t-test
	hLRRK2*G2019S: Young adults vs Aged	t=2.648	p=0.0129	Unpaired two-tailed t-test
4C	Young adults: WT vs hLRRK2*G2019S	t=0.3755	p=0.7093	Unpaired two-tailed t-test
	Aged: WT vs hLRRK2*G2019S	t=3.469	p=0.0019	Unpaired two-tailed t-test
	WT: Young adults vs Aged	t=2.693	p=0.0108	Unpaired two-tailed t-test
	hLRRK2*G2019S: Young adults vs Aged	t=1.144	p=0.2620	Unpaired two-tailed t-test
4D	Young adults: WT vs hLRRK2*G2019S	t=0.7226	p=0.4742	Unpaired two-tailed t-test
	Aged: WT vs hLRRK2*G2019S	t=3.481	p=0.0019	Unpaired two-tailed t-test
	WT: Young adults vs Aged	t=1.861	p=0.0711	Unpaired two-tailed t-test
	hLRRK2*G2019S: Young adults vs Aged	t=2.647	p=0.0130	Unpaired two-tailed t-test
4E	Young adults: WT vs hLRRK2*G2019S	t=0.4846	p=0.6307	Unpaired two-tailed t-test
	Aged: WT vs hLRRK2*G2019S	t=2.589	p=0.0173	Unpaired two-tailed t-test with Welch's correction
	WT: Young adults vs Aged	t=0.3882	p=0.7002	Unpaired two-tailed t-test
	hLRRK2*G2019S: Young adults vs Aged	t=2.702	p=0.0114	Unpaired two-tailed t-test
4F	Young adults: WT vs hLRRK2*G2019S	t=0.05164	p=0.9591	Unpaired two-tailed t-test
	Aged: WT vs hLRRK2*G2019S	t=1.727	p=0.1000	Unpaired two-tailed t-test with Welch's correction
	WT: Young adults vs Aged	t=0.07669	p=0.9393	Unpaired two-tailed t-test
	hLRRK2*G2019S: Young adults vs Aged	t=1.811	p=0.0806	Unpaired two-tailed t-test
4G	Young adults: WT vs hLRRK2*G2019S	t=1.161	p=0.2526	Unpaired two-tailed t-test
	Aged: WT vs hLRRK2*G2019S	t=2.592	p=0.0187	Unpaired two-tailed t-test with Welch's correction
	WT: Young adults vs Aged	t=1.205	p=0.2361	Unpaired two-tailed t-test
	hLRRK2*G2019S: Young adults vs Aged	t=2.522	p=0.0211	Unpaired two-tailed t-test with Welch's correction
4H	Young adults: WT vs hLRRK2*G2019S	t=1.144	p=0.2595	Unpaired two-tailed t-test
	Aged: WT vs hLRRK2*G2019S	t=2.355	p=0.0267	Unpaired two-tailed t-test
	WT: Young adults vs Aged	t=0.02952	p=0.9766	Unpaired two-tailed t-test
	hLRRK2*G2019S: Young adults vs Aged	t=1.378	p=0.1788	Unpaired two-tailed t-test
4I	Young adults: WT vs hLRRK2*G2019S	t=2.402	p=0.0212	Unpaired two-tailed t-test
	Aged: WT vs hLRRK2*G2019S	t=1.743	p=0.0947	Unpaired two-tailed t-test
	WT: Young adults vs Aged	t=1.346	p=0.1869	Unpaired two-tailed t-test
	hLRRK2*G2019S: Young adults vs Aged	t=3.040	p=0.0052	Unpaired two-tailed t-test
4J	Young adults: WT vs hLRRK2*G2019S	t=1.140	p=0.2583	Unpaired two-tailed t-test
	Aged: WT vs hLRRK2*G2019S	t=1.311	p=0.1990	Unpaired two-tailed t-test
	WT: Young adults vs Aged	t=2.346	p=0.0229	Unpaired two-tailed t-test
	hLRRK2*G2019S: Young adults vs Aged	t=4.828	p<0.0001	Unpaired two-tailed t-test
4K	Young adults: WT vs hLRRK2*G2019S	t=1.560	p=0.1249	Unpaired two-tailed t-test with Welch's correction
	Aged: WT vs hLRRK2*G2019S	t=0.4585	p=0.6496	Unpaired two-tailed t-test
	WT: Young adults vs Aged	t=1.438	p=0.1564	Unpaired two-tailed t-test
	hLRRK2*G2019S: Young adults vs Aged	t=0.5266	p=0.6009	Unpaired two-tailed t-test
4L	Young adults: WT vs hLRRK2*G2019S	t=1.520	p=0.1332	Unpaired two-tailed t-test
	Aged: WT vs hLRRK2*G2019S	t=1.261	p=0.2162	Unpaired two-tailed t-test
	WT: Young adults vs Aged	t=1.683	p=0.1080	Unpaired two-tailed t-test with Welch's correction
	hLRRK2*G2019S: Young adults vs Aged	t=4.976	p<0.0001	Unpaired two-tailed t-test
4M	Young adults: WT vs hLRRK2*G2019S	t=2.267	p=0.0281	Unpaired two-tailed t-test with Welch's correction
	Aged: WT vs hLRRK2*G2019S	t=0.9488	p=0.3496	Unpaired two-tailed t-test
	WT: Young adults vs Aged	t=1.518	p=0.1454	Unpaired two-tailed t-test with Welch's correction
	hLRRK2*G2019S: Young adults vs Aged	t=1.050	p=0.2991	Unpaired two-tailed t-test

Statistical report: Figure 5.

Figure 5				
**Figure**	**Experimental groups compared**	**Test statistic**	**P value**	**Statistical test**
5B	Young adults: WT vs hLRRK2*G2019S	t=3.636	p=0.0019	Unpaired two-tailed t-test
	Aged: WT vs hLRRK2*G2019S	t=1.450	p=0.1723	Unpaired two-tailed t-test with Welch's correction
	WT: Young adults vs Aged	t=5.720	p=0.0001	Unpaired two-tailed t-test with Welch's correction
	hLRRK2*G2019S: Young adults vs Aged	t=4.896	p=0.0002	Unpaired two-tailed t-test
5D	Young adults: WT vs hLRRK2*G2019S	t=4.181	p=0.0015	Unpaired two-tailed t-test with Welch's correction
	Aged: WT vs hLRRK2*G2019S	t=0.1597	p=0.8749	Unpaired two-tailed t-test
	WT: Young adults vs Aged	t=6.860	p<0.0001	Unpaired two-tailed t-test with Welch's correction
	hLRRK2*G2019S: Young adults vs Aged	t=4.392	p=0.0005	Unpaired two-tailed t-test
5F	Young adults: WT vs hLRRK2*G2019S	t=1.013	p=0.3227	Unpaired two-tailed t-test
	Aged: WT vs hLRRK2*G2019S	t=0.8908	p=0.3892	Unpaired two-tailed t-test
	WT: Young adults vs Aged	t=4.732	p=0.0001	Unpaired two-tailed t-test
	hLRRK2*G2019S: Young adults vs Aged	t=2.170	p=0.0491	Unpaired two-tailed t-test
5H	Young adults: WT vs hLRRK2*G2019S	t=1.189	p=0.2476	Unpaired two-tailed t-test
	Aged: WT vs hLRRK2*G2019S	t=0.1076	p=0.9160	Unpaired two-tailed t-test
	WT: Young adults vs Aged	t=1.938	p=0.0875	Unpaired two-tailed t-test with Welch's correction
	hLRRK2*G2019S: Young adults vs Aged	t=3.228	p=0.0066	Unpaired two-tailed t-test
5I	Young adults: WT vs hLRRK2*G2019S	t=2.289	p=0.0428	Unpaired two-tailed t-test
	Aged: WT vs hLRRK2*G2019S	U=11	p=0.1807	Mann–Whitney U test
	WT: Young adults vs Aged	t=1.750	p=0.1056	Unpaired two-tailed t-test
	hLRRK2*G2019S: Young adults vs Aged	t=8.780	p<0.0001	Unpaired two-tailed t-test
5J	Young adults: WT vs hLRRK2*G2019S	t=0.5917	p=0.5660	Unpaired two-tailed t-test
	Aged: WT vs hLRRK2*G2019S	t=0.4951	p=0.6303	Unpaired two-tailed t-test
	WT: Young adults vs Aged	t=8.070	p<0.0001	Unpaired two-tailed t-test
	hLRRK2*G2019S: Young adults vs Aged	t=13.66	p<0.0001	Unpaired two-tailed t-test
5K	Young adults: WT vs hLRRK2*G2019S	t=1.411	p=0.1859	Unpaired two-tailed t-test
	Aged: WT vs hLRRK2*G2019S	t=3.382	p=0.0061	Unpaired two-tailed t-test
	WT: Young adults vs Aged	t=0.4593	p=0.6543	Unpaired two-tailed t-test
	hLRRK2*G2019S: Young adults vs Aged	t=4.502	p=0.0011	Unpaired two-tailed t-test
5L	Young adults: WT vs hLRRK2*G2019S	t=2.105	p=0.0591	Unpaired two-tailed t-test
	Aged: WT vs hLRRK2*G2019S	t=1.094	p=0.2973	Unpaired two-tailed t-test
	WT: Young adults vs Aged	t=3.044	p=0.0194	Unpaired two-tailed t-test with Welch's correction
	hLRRK2*G2019S: Young adults vs Aged	t=2.261	p=0.0473	Unpaired two-tailed t-test
5M	Young adults: WT vs hLRRK2*G2019S	t=0.9684	p=0.3536	Unpaired two-tailed t-test
	Aged: WT vs hLRRK2*G2019S	t=1.158	p=0.2713	Unpaired two-tailed t-test
	WT: Young adults vs Aged	t=4.946	p=0.0003	Unpaired two-tailed t-test
	hLRRK2*G2019S: Young adults vs Aged	t=4.184	p=0.0080	Unpaired two-tailed t-test with Welch's correction
5N	Young adults: WT vs hLRRK2*G2019S	t=2.272	p=0.0442	Unpaired two-tailed t-test
	Aged: WT vs hLRRK2*G2019S	t=1.023	p=0.3285	Unpaired two-tailed t-test
	WT: Young adults vs Aged	t=2.2363	p=0.0358	Unpaired two-tailed t-test
	hLRRK2*G2019S: Young adults vs Aged	t=4.401	p=0.0013	Unpaired two-tailed t-test
5O	Young adults: WT vs hLRRK2*G2019S	t=0.4052	p=0.6931	Unpaired two-tailed t-test
	Aged: WT vs hLRRK2*G2019S	t=0.1491	p=0.8841	Unpaired two-tailed t-test
	WT: Young adults vs Aged	t=3.641	p=0.0034	Unpaired two-tailed t-test
	hLRRK2*G2019S: Young adults vs Aged	t=3.080	p=0.0116	Unpaired two-tailed t-test
5P	Young adults: WT vs hLRRK2*G2019S	U=0	p=0.0012	Mann–Whitney U test
	Aged: WT vs hLRRK2*G2019S	t=0.7554	p=0.4659	Unpaired two-tailed t-test
	WT: Young adults vs Aged	U=11.50	p=0.1043	Mann–Whitney U test
	hLRRK2*G2019S: Young adults vs Aged	t=1.753	p=0.1101	Unpaired two-tailed t-test

Statistical report: Figures 6-7.

Figure 6					
Figure	Experimental groups compared	Test statistic	P value	Post hoc P values	Statistical test
6B	Saline: WT vs hLRRK2*G2019S	t=0.6066	p=0.5506		Unpaired two-tailed t-test
	MPTP 20mg/kg 1 month: WT vs hLRRK2*G2019S	t=1.478	p=0.1675		Unpaired two-tailed t-test
	MPTP 20mg/kg 3 months: WT vs hLRRK2*G2019S	t=1.554	p=0.1588		Unpaired two-tailed t-test
	WT: Saline *vs* MPTP 20mg/kg (1 month, 3 months)	F*(2.000,6.398)=155.2	p<0.0001	p<0.0001, p=0.0002	Brown-Forsythe and Welch ANOVA test (Dunnett's T3 post hoc test)
	hLRRK2*G2019S: Saline *vs* MPTP 20mg/kg (1 month, 3 months)	F(2,15)=130.5	p<0.0001	p<0.0001, p<0.0001	ANOVA test (Dunnett's post hoc test)
6C	Saline: WT vs hLRRK2*G2019S	t=0.9463	p=0.3663		Unpaired two-tailed t-test
	MPTP 5mg/kg 1 month: WT vs hLRRK2*G2019S	t=0.2240	p=0.8262		Unpaired two-tailed t-test
	WT: Saline *vs* MPTP 5mg/kg (1 month)	t=0.1023	p=0.9202		Unpaired two-tailed t-test
	hLRRK2*G2019S: Saline vs MPTP 5mg/kg (1 month)	t=1.457	p=0.1730		Unpaired two-tailed t-test
6E	Saline: WT vs hLRRK2*G2019S	t=0.2654	p=0.7933		Unpaired two-tailed t-test
	MPTP 20mg/kg 1 month: WT vs hLRRK2*G2019S	t=0.4505	p=0.6611		Unpaired two-tailed t-test
	MPTP 20mg/kg 3 months: WT vs hLRRK2*G2019S	t=1.060	p=0.3201		Unpaired two-tailed t-test
	WT: Saline vs MPTP 20mg/kg (1 month, 3 months)	F(2,25)=50.50	p<0.0001	p<0.0001, p<0.0001	ANOVA test (Dunnett's post hoc test)
	hLRRK2*G2019S: Saline vs MPTP 20mg/kg (1 month, 3 months)	F(2,15)=51.24	p<0.0001	p<0.0001, p<0.0001	ANOVA test (Dunnett's post hoc test)
6F	Saline: WT vs hLRRK2*G2019S	t=0.4455	p=0.6646		Unpaired two-tailed t-test
	MPTP 5mg/kg 1 month: WT vs hLRRK2*G2019S	t=0.2187	p=0.8305		Unpaired two-tailed t-test
	WT: Saline vs MPTP 5mg/kg (1 month)	t=0.0490	p=0.9618		Unpaired two-tailed t-test
	hLRRK2*G2019S: Saline vs MPTP 5mg/kg (1 month)	t=0.1169	p=0.9091		Unpaired two-tailed t-test
6G	Saline: WT vs hLRRK2*G2019S	t=0.05038	p=0.9601		Unpaired two-tailed t-test
	MPTP 20mg/kg 1 month: WT vs hLRRK2*G2019S	t=1.359	p=0.1919		Unpaired two-tailed t-test with Welch's correction
	MPTP 20mg/kg 3 months: WT vs hLRRK2*G2019S	t=0.2913	p=0.7748		Unpaired two-tailed t-test
	WT: Saline vs MPTP 20mg/kg (1 month, 3 months)	W(2.000,29.55)=303.0	p<0.0001	p<0.0001, p<0.0001	Brown-Forsythe and Welch ANOVA test (Dunnett's T3 post hoc test)
	hLRRK2*G2019S: Saline vs MPTP 20mg/kg (1 month, 3 months)	H=20.91	p<0.0001	p=0.0003, p=0.0003	Kruskal-Wallis test (Dunn's post hoc test)
6H	Saline: WT vs hLRRK2*G2019S	t=1.578	p=0.1776		Unpaired two-tailed t-test with Welch's correction
	MPTP 5mg/kg 1 month: WT vs hLRRK2*G2019S	t=1.883	p=0.0822		Unpaired two-tailed t-test
	WT: Saline vs MPTP 5mg/kg (1 month)	t=0.7942	p=0.4439		Unpaired two-tailed t-test
	hLRRK2*G2019S: Saline vs MPTP 5mg/kg (1 month)	t=0.2214	p=0.8308		Unpaired two-tailed t-test with Welch's correction
6I	Saline: WT vs hLRRK2*G2019S	t=0.03573	p=0.9717		Unpaired two-tailed t-test
	MPTP 20mg/kg 1 month: WT vs hLRRK2*G2019S	t=0.8356	p=0.4130		Unpaired two-tailed t-test with Welch's correction
	MPTP 20mg/kg 3 months: WT vs hLRRK2*G2019S	t=0.4202	p=0.6803		Unpaired two-tailed t-test
	WT: Saline vs MPTP 20mg/kg (1 month, 3 months)	W(2.000,29.61)=55.42	p<0.0001	p<0.0001, p<0.0001	Brown-Forsythe and Welch ANOVA test (Dunnett's T3 post hoc test)
	hLRRK2*G2019S: Saline vs MPTP 20mg/kg (1 month, 3 months)	H=20.87	p<0.0001	p=0.0003, p=0.0003	Kruskal-Wallis test (Dunn's post hoc test)
6J	Saline: WT vs hLRRK2*G2019S	t=0.4294	p=0.6777		Unpaired two-tailed t-test
	MPTP 5mg/kg 1 month: WT vs hLRRK2*G2019S	t=1.438	p=0.1740		Unpaired two-tailed t-test
	WT: Saline vs MPTP 5mg/kg (1 month)	t=1.234	p=0.2428		Unpaired two-tailed t-test
	hLRRK2*G2019S: Saline vs MPTP 5mg/kg (1 month)	t=0.5064	p=0.6226		Unpaired two-tailed t-test
6K	Saline: WT vs hLRRK2*G2019S	t=0.1728	p=0.8639		Unpaired two-tailed t-test
	MPTP 20mg/kg 1 month: WT vs hLRRK2*G2019S	t=0.3562	p=0.7251		Unpaired two-tailed t-test
	MPTP 20mg/kg 3 months: WT vs hLRRK2*G2019S	t=0.5053	p=0.6207		Unpaired two-tailed t-test
	WT: Saline vs MPTP 20mg/kg (1 month, 3 months)	F(2,43)=20.21	p<0.0001	p=0.0001, p<0.0001	ANOVA test (Dunnett's post hoc test)
	hLRRK2*G2019S: Saline vs MPTP 20mg/kg (1 month, 3 months)	F(2,26)=17.16	p<0.0001	p=0.0008, p<0.0001	ANOVA test (Dunnett's post hoc test)
6L	Saline: WT vs hLRRK2*G2019S	t=0.03110	p=0.9759		Unpaired two-tailed t-test
	MPTP 5mg/kg 1 month: WT vs hLRRK2*G2019S	t=1.472	p=0.1745		Unpaired two-tailed t-test with Welch's correction
	WT: Saline vs MPTP 5mg/kg (1 month)	t=1.301	p=0.2199		Unpaired two-tailed t-test
	hLRRK2*G2019S: Saline vs MPTP 5mg/kg (1 month)	t=0.3572	p=0.7277		Unpaired two-tailed t-test
6M	Saline: WT vs hLRRK2*G2019S	t=0.1071	p=0.9154		Unpaired two-tailed t-test
	MPTP 20mg/kg 1 month: WT vs hLRRK2*G2019S	t=0.1358	p=0.8933		Unpaired two-tailed t-test with Welch's correction
	MPTP 20mg/kg 3 months: WT vs hLRRK2*G2019S	t=0.1268	p=0.9008		Unpaired two-tailed t-test
	WT: Saline vs MPTP 20mg/kg (1 month, 3 months)	W(2.000,16.82)=26.62	p<0.0001	p<0.0001, p=0.0005	Brown-Forsythe and Welch ANOVA test (Dunnett's T3 post hoc test)
	hLRRK2*G2019S: Saline vs MPTP 20mg/kg (1 month, 3 months)	F(2,26)=41.38	p<0.0001	p<0.0001, p<0.0001	ANOVA test (Dunnett's post hoc test)
6N	Saline: WT vs hLRRK2*G2019S	t=1.425	p=0.1879		Unpaired two-tailed t-test
	MPTP 5mg/kg 1 month: WT vs hLRRK2*G2019S	t=0.8845	p=0.3925		Unpaired two-tailed t-test
	WT: Saline vs MPTP 5mg/kg (1 month)	t=1.151	p=0.2742		Unpaired two-tailed t-test
	hLRRK2*G2019S: Saline vs MPTP 5mg/kg (1 month)	t=0.7068	p=0.5049		Unpaired two-tailed t-test with Welch's correction

Figure 7					
Figure	Experimental groups compared	Test statistic	P value	Post hoc P values	Statistical test
7B	Saline: WT vs hLRRK2*G2019S	t=2.443	p=0.0326		Unpaired two-tailed t-test
	MPTP 20mg/kg 1 month: WT vs hLRRK2*G2019S	t=0.7805	p=0.4502		Unpaired two-tailed t-test
	MPTP 20mg/kg 3 months: WT vs hLRRK2*G2019S	t=0.7301	p=0.4806		Unpaired two-tailed t-test
	WT: Saline vs MPTP 20mg/kg (1 month, 3 months)	F(2,19)=34.46	p<0.0001	p<0.0001, p<0.0001	ANOVA test (Dunnett's post hoc test)
	hLRRK2*G2019S: Saline vs MPTP 20mg/kg (1 month, 3 months)	F(2,15)=11.54	p=0.0009	p=0.0025, p=0.0011	ANOVA test (Dunnett's post hoc test)
7C	Saline: WT vs hLRRK2*G2019S	t=8.849	p<0.0001		Unpaired two-tailed t-test
	MPTP 5mg/kg 1 month: WT vs hLRRK2*G2019S	t=2.641	p=0.0203		Unpaired two-tailed t-test
	WT: Saline vs MPTP 5mg/kg (1 month)	t=3.870	p=0.0022		Unpaired two-tailed t-test
	hLRRK2*G2019S: Saline vs MPTP 5mg/kg (1 month)	t=1.199	p=0.2556		Unpaired two-tailed t-test
7E	Saline: WT vs hLRRK2*G2019S	t=2.564	p=0.0282		Unpaired two-tailed t-test
	MPTP 20mg/kg 1 month: WT vs hLRRK2*G2019S	t=0.5140	p=0.6174		Unpaired two-tailed t-test
	MPTP 20mg/kg 3 months: WT vs hLRRK2*G2019S	t=2.789	p=0.0164		Unpaired two-tailed t-test
	WT: Saline vs MPTP 20mg/kg (1 month, 3 months)	F(2,20)=43.62	p<0.0001	p<0.0001, p<0.0001	ANOVA test (Dunnett's post hoc test)
	hLRRK2*G2019S: Saline vs MPTP 20mg/kg (1 month, 3 months)	F(2,13)=12.07	p=0.0011	p=0.0006, p=0.0110	ANOVA test (Dunnett's post hoc test)
7F	Saline: WT vs hLRRK2*G2019S	t=6.913	p<0.0001		Unpaired two-tailed t-test
	MPTP 5mg/kg 1 month: WT vs hLRRK2*G2019S	t=3.248	p=0.0063		Unpaired two-tailed t-test
	WT: Saline vs MPTP 5mg/kg (1 month)	t=6.925	p<0.0001		Unpaired two-tailed t-test
	hLRRK2*G2019S: Saline vs MPTP 5mg/kg (1 month)	t=2.803	p=0.0172		Unpaired two-tailed t-test
7H	Saline: WT vs hLRRK2*G2019S	t=1.251	p=0.2288		Unpaired two-tailed t-test
	MPTP 20mg/kg 1 month: WT vs hLRRK2*G2019S	t=0.6103	p=0.5541		Unpaired two-tailed t-test
	MPTP 20mg/kg 3 months: WT vs hLRRK2*G2019S	t=2.722	p=0.0199		Unpaired two-tailed t-test
	WT: Saline vs MPTP 20mg/kg (1 month, 3 months)	F(2,25)=36.26	p<0.0001	p<0.0001, p=0.0011	ANOVA test (Dunnett's post hoc test)
	hLRRK2*G2019S: Saline vs MPTP 20mg/kg (1 month, 3 months)	F(2,13)=17.88	p=0.0002	p=0.0002, p=0.8703	ANOVA test (Dunnett's post hoc test)
7I	Saline: WT vs hLRRK2*G2019S	t=0.7786	p=0.4542		Unpaired two-tailed t-test
	MPTP 5mg/kg 1 month: WT vs hLRRK2*G2019S	t=1.101	p=0.2907		Unpaired two-tailed t-test
	WT: Saline vs MPTP 5mg/kg (1 month)	t=0.2102	p=0.8371		Unpaired two-tailed t-test
	hLRRK2*G2019S: Saline vs MPTP 5mg/kg (1 month)	t=0.7540	p=0.4667		Unpaired two-tailed t-test
7K	Saline: WT vs hLRRK2*G2019S	t=1.109	p=0.2849		Unpaired two-tailed t-test
	MPTP 20mg/kg 1 month: WT vs hLRRK2*G2019S	t=2.591	p=0.0251		Unpaired two-tailed t-test
	MPTP 20mg/kg 3 months: WT vs hLRRK2*G2019S	t=1.165	p=0.2687		Unpaired two-tailed t-test
	WT: Saline vs MPTP 20mg/kg (1 month, 3 months)	F(2,25)=35.74	p<0.0001	p<0.0001, p<0.0001	ANOVA test (Dunnett's post hoc test)
	hLRRK2*G2019S: Saline vs MPTP 20mg/kg (1 month, 3 months)	F(2,12)=37.47	p<0.0001	p<0.0001, p=0.0015	ANOVA test (Dunnett's post hoc test)
7L	Saline: WT vs hLRRK2*G2019S	t=0.9458	p=0.3665		Unpaired two-tailed t-test
	MPTP 5mg/kg 1 month: WT vs hLRRK2*G2019S	t=0.4429	p=0.6651		Unpaired two-tailed t-test
	WT: Saline vs MPTP 5mg/kg (1 month)	t=0.9919	p=0.3408		Unpaired two-tailed t-test
	hLRRK2*G2019S: Saline vs MPTP 5mg/kg (1 month)	t=1.318	p=0.2144		Unpaired two-tailed t-test

Statistical report: Figure 8.

Figure 8					
**Figure**	**Experimental groups compared**	**Test statistic**	**P value**	**Post hoc P values**	**Statistical test**
8A	MPTP 20mg/kg 1 month: WT vs hLRRK2*G2019S	t=3.667	p=0.0053		Unpaired two-tailed t-test with Welch's correction
	MPTP 20mg/kg 3 months: WT vs hLRRK2*G2019S	t=0.9050	p=0.3819		Unpaired two-tailed t-test
	WT: Saline vs MPTP 20mg/kg (1 month, 3 months)	F(2,20)=1.914	p=0.1736	p=0.5712, p=0.5149	ANOVA test (Dunnett's post hoc test)
	hLRRK2*G2019S: Saline vs MPTP 20mg/kg (1 month, 3 months)	W(2.000,11.67)=4.926	p=0.0281	p=0.1674, p=0.9975	Brown-Forsythe and Welch ANOVA test (Dunnett's T3 post hoc test)
8B	MPTP 5mg/kg 1 month: WT vs hLRRK2*G2019S	t=1.668	p=0.1191		Unpaired two-tailed t-test
	WT: Saline vs MPTP 5mg/kg (1 month)	t=0.06512	p=0.9491		Unpaired two-tailed t-test
	hLRRK2*G2019S: Saline vs MPTP 5mg/kg (1 month)	t=1.451	p=0.1746		Unpaired two-tailed t-test
8C	MPTP 20mg/kg 1 month: WT vs hLRRK2*G2019S	t=1.228	p=0.2429		Unpaired two-tailed t-test
	MPTP 20mg/kg 3 months: WT vs hLRRK2*G2019S	t=0.2005	p=0.8448		Unpaired two-tailed t-test
	WT: Saline vs MPTP 20mg/kg (1 month, 3 months)	F(2,18)=0.1030	p=0.9027	p>0.9999, p=0.8963	ANOVA test (Dunnett's post hoc test)
	hLRRK2*G2019S: Saline vs MPTP 20mg/kg (1 month, 3 months)	F(2,18)=1.203	p=0.3234	p=0.6050, p=0.6784	ANOVA test (Dunnett's post hoc test)
8D	MPTP 5mg/kg 1 month: WT vs hLRRK2*G2019S	t=1.372	p=0.1973		Unpaired two-tailed t-test
	WT: Saline vs MPTP 5mg/kg (1 month)	t=0.7281	p=0.4928		Unpaired two-tailed t-test with Welch's correction
	hLRRK2*G2019S: Saline vs MPTP 5mg/kg (1 month)	t=0.09785	p=0.9242		Unpaired two-tailed t-test
8E	MPTP 20mg/kg 1 month: WT vs hLRRK2*G2019S	t=0.5601	p=0.5857		Unpaired two-tailed t-test
	MPTP 20mg/kg 3 months: WT vs hLRRK2*G2019S	t=1.647	p=0.1581		Unpaired two-tailed t-test with Welch's correction
	WT: Saline vs MPTP 20mg/kg (1 month, 3 months)	F(2,17)=1.355	p=0.2844	p=0.7736, p=0.2100	ANOVA test (Dunnett's post hoc test)
	hLRRK2*G2019S: Saline vs MPTP 20mg/kg (1 month, 3 months)	H=3.492	p=0.1793	p=0.1233, p=0.9095	Kruskal-Wallis test (Dunn's post hoc test)
8F	MPTP 5mg/kg 1 month: WT vs hLRRK2*G2019S	t=0.4113	p=0.6922		Unpaired two-tailed t-test with Welch's correction
	WT: Saline vs MPTP 5mg/kg (1 month)	t=0.6433	p=0.5321		Unpaired two-tailed t-test
	hLRRK2*G2019S: Saline vs MPTP 5mg/kg (1 month)	t=0.02052	p=0.9841		Unpaired two-tailed t-test
8G	MPTP 20mg/kg 1 month: WT vs hLRRK2*G2019S	t=1.942	p=0.0760		Unpaired two-tailed t-test
	MPTP 20mg/kg 3 months: WT vs hLRRK2*G2019S	t=0.8410	p=0.4200		Unpaired two-tailed t-test
	WT: Saline vs MPTP 20mg/kg (1 month, 3 months)	F(2,17)=0.3384	p=0.7176	p=0.7292; p=0.9940	ANOVA test (Dunnett's post hoc test)
	hLRRK2*G2019S: Saline vs MPTP 20mg/kg (1 month, 3 months)	F(2,18)=4.994	p=0.0188	p=0.0122; p=0.6847	ANOVA test (Dunnett's post hoc test)
8H	MPTP 5mg/kg 1 month: WT vs hLRRK2*G2019S	t=3.915	p=0.0091		Unpaired two-tailed t-test with Welch's correction
	WT: Saline vs MPTP 5mg/kg (1 month)	t=0.1090	p=0.9150		Unpaired two-tailed t-test
	hLRRK2*G2019S: Saline vs MPTP 5mg/kg (1 month)	t=3.962	p=0.0085		Unpaired two-tailed t-test with Welch's correction
8I	MPTP 20mg/kg 1 month: WT vs hLRRK2*G2019S	U=6	p=0.0200		Mann-Whitney U test
	MPTP 20mg/kg 3 months: WT vs hLRRK2*G2019S	t=3.721	p=0.0029		Unpaired two-tailed t-test
	WT: Saline vs MPTP 20mg/kg (1 month, 3 months)	F(2,20)=1.695	p=0.2089	p=0.1996, p=0.2461	ANOVA test (Dunnett's post hoc test)
	hLRRK2*G2019S: Saline vs MPTP 20mg/kg (1 month, 3 months)	H=1.859	p=0.4087	p>0.9999, p=0.3826	Kruskal-Wallis test (Dunn's post hoc test)
8J	MPTP 5mg/kg 1 month: WT vs hLRRK2*G2019S	t=0.8486	p=0.4160		Unpaired two-tailed t-test
	WT: Saline vs MPTP 5mg/kg (1 month)	t=0.1447	p=0.8881		Unpaired two-tailed t-test
	hLRRK2*G2019S: Saline vs MPTP 5mg/kg (1 month)	t=0.7353	p=0.4775		Unpaired two-tailed t-test
8K	MPTP 20mg/kg 1 month: WT vs hLRRK2*G2019S	t=1.042	p=0.3180		Unpaired two-tailed t-test
	MPTP 20mg/kg 3 months: WT vs hLRRK2*G2019S	t=3.019	p=0.0107		Unpaired two-tailed t-test
	WT: Saline vs MPTP 20mg/kg (1 month, 3 months)	F(2,19)=4.627	p=0.0231	p=0.1515, p=0.0129	ANOVA test (Dunnett's post hoc test)
	hLRRK2*G2019S: Saline vs MPTP 20mg/kg (1 month, 3 months)	F(2,18)=0.3312	p=0.7223	p=0.6500, p=0.8506	ANOVA test (Dunnett's post hoc test)
8L	MPTP 5mg/kg 1 month: WT vs hLRRK2*G2019S	t=0.2459	p=0.8099		Unpaired two-tailed t-test
	WT: Saline vs MPTP 5mg/kg (1 month)	t=1.701	p=0.1170		Unpaired two-tailed t-test
	hLRRK2*G2019S: Saline vs MPTP 5mg/kg (1 month)	t=1.219	p=0.2507		Unpaired two-tailed t-test
8M	MPTP 20mg/kg 1 month: WT vs hLRRK2*G2019S	t=3.193	p=0.0077		Unpaired two-tailed t-test
	MPTP 20mg/kg 3 months: WT vs hLRRK2*G2019S	t=0.08786	p=0.9322		Unpaired two-tailed t-test with Welch's correction
	WT: Saline vs MPTP 20mg/kg (1 month, 3 months)	F(2,19)=0.4230	p=0.6611	p=0.5633, p=0.8549	ANOVA test (Dunnett's post hoc test)
	hLRRK2*G2019S: Saline vs MPTP 20mg/kg (1 month, 3 months)	F(2,18)=4.431	p=0.0272	p=0.0462, p=0.8812	ANOVA test (Dunnett's post hoc test)
8N	MPTP 5mg/kg 1 month: WT vs hLRRK2*G2019S	t=1.479	p=0.1699		Unpaired two-tailed t-test
	WT: Saline vs MPTP 5mg/kg (1 month)	t=0.4522	p=0.6618		Unpaired two-tailed t-test
	hLRRK2*G2019S: Saline vs MPTP 5mg/kg (1 month)	t=0.8403	p=0.4186		Unpaired two-tailed t-test
8O	MPTP 20mg/kg 1 month: WT vs hLRRK2*G2019S	t=0.4043	p=0.6931		Unpaired two-tailed t-test
	MPTP 20mg/kg 3 months: WT vs hLRRK2*G2019S	t=1.600	p=0.1356		Unpaired two-tailed t-test
	WT: Saline vs MPTP 20mg/kg (1 month, 3 months)	F(2,20)=1.230	p=0.3136	p=0.2272; p=0.6505	ANOVA test (Dunnett's post hoc test)
	hLRRK2*G2019S: Saline vs MPTP 20mg/kg (1 month, 3 months)	F(2,18)=3.173	p=0.0660	p=0.1561; p=0.6726	ANOVA test (Dunnett's post hoc test)
8P	MPTP 5mg/kg 1 month: WT vs hLRRK2*G2019S	t=0.6034	p=0.5566		Unpaired two-tailed t-test
	WT: Saline vs MPTP 5mg/kg (1 month)	t=0.7387	p=0.4743		Unpaired two-tailed t-test
	hLRRK2*G2019S: Saline vs MPTP 5mg/kg (1 month)	t=0.2933	p=0.7748		Unpaired two-tailed t-test

Statistical report: Supplementary Figure 1.

Supplementary Figure 1				
**Figure**	**Experimental groups compared**	**Test statistic**	**P value**	**Statistical test**
Supp.Fig 1A	Young adults: WT vs hLRRK2*G2019S	t=0.9056	p=0.3846	Unpaired two-tailed t-test
	Aged: WT vs hLRRK2*G2019S	t=1.279	p=0.2271	Unpaired two-tailed t-test
	WT: Young adults vs Aged	t=2.644	p=0.0352	Unpaired two-tailed t-test with Welch's correction
	hLRRK2*G2019S: Young adults vs Aged	t=4.883	p=0.0006	Unpaired two-tailed t-test
Supp.Fig 1B	Young adults: WT vs hLRRK2*G2019S	t=1.543	p=0.1755	Unpaired two-tailed t-test with Welch's correction
	Aged: WT vs hLRRK2*G2019S	t=2.178	p=0.0723	Unpaired two-tailed t-test with Welch's correction
	WT: Young adults vs Aged	t=0.6760	p=0.5128	Unpaired two-tailed t-test
	hLRRK2*G2019S: Young adults vs Aged	t=0.5458	p=0.5972	Unpaired two-tailed t-test
Supp.Fig 1C	Young adults: WT vs hLRRK2*G2019S	t=0.2186	p=0.8310	Unpaired two-tailed t-test
	Aged: WT vs hLRRK2*G2019S	t=2.312	p=0.0412	Unpaired two-tailed t-test
	WT: Young adults vs Aged	t=0.7040	p=0.4949	Unpaired two-tailed t-test
	hLRRK2*G2019S: Young adults vs Aged	U=5	p=0.0411	Mann–Whitney U test
Supp.Fig 1D	Young adults: WT vs hLRRK2*G2019S	t=0.8804	p=0.3975	Unpaired two-tailed t-test
	Aged: WT vs hLRRK2*G2019S	t=1.110	p=0.2909	Unpaired two-tailed t-test
	WT: Young adults vs Aged	t=2.172	p=0.0666	Unpaired two-tailed t-test with Welch's correction
	hLRRK2*G2019S: Young adults vs Aged	t=4.067	p=0.0068	Unpaired two-tailed t-test with Welch's correction
Supp.Fig 1E	Young adults: WT vs hLRRK2*G2019S	t=0.6134	p=0.5521	Unpaired two-tailed t-test
	Aged: WT vs hLRRK2*G2019S	t=1.010	p=0.3340	Unpaired two-tailed t-test
	WT: Young adults vs Aged	U=3	p=0.0041	Mann–Whitney U test
	hLRRK2*G2019S: Young adults vs Aged	t=7.087	p<0.0001	Unpaired two-tailed t-test
Supp.Fig 1F	Young adults: WT vs hLRRK2*G2019S	t=2.773	p=0.0181	Unpaired two-tailed t-test
	Aged: WT vs hLRRK2*G2019S	t=1.013	p=0.3327	Unpaired two-tailed t-test
	WT: Young adults vs Aged	t=5.172	p=0.0017	Unpaired two-tailed t-test with Welch's correction
	hLRRK2*G2019S: Young adults vs Aged	t=5.514	p=0.0025	Unpaired two-tailed t-test with Welch's correction
Supp.Fig 1G	Young adults: WT vs hLRRK2*G2019S	t=0.2534	p=0.8046	Unpaired two-tailed t-test
	Aged: WT vs hLRRK2*G2019S	t=0.9549	p=0.3602	Unpaired two-tailed t-test
	WT: Young adults vs Aged	t=1.833	p=0.0917	Unpaired two-tailed t-test
	hLRRK2*G2019S: Young adults vs Aged	t=1.053	p=0.3173	Unpaired two-tailed t-test
Supp.Fig 1H	Young adults: WT vs hLRRK2*G2019S	t=0.8050	p=0.4379	Unpaired two-tailed t-test
	Aged: WT vs hLRRK2*G2019S	t=0.01745	p=0.9864	Unpaired two-tailed t-test
	WT: Young adults vs Aged	t=1.799	p=0.1125	Unpaired two-tailed t-test with Welch's correction
	hLRRK2*G2019S: Young adults vs Aged	t=2.789	p=0.0191	Unpaired two-tailed t-test
Supp.Fig 1I	Young adults: WT vs hLRRK2*G2019S	U=16	p=0.8182	Mann–Whitney U test
	Aged: WT vs hLRRK2*G2019S	t=0.4875	p=0.6376	Unpaired two-tailed t-test
	WT: Young adults vs Aged	U=15	p=0.6991	Mann–Whitney U test
	hLRRK2*G2019S: Young adults vs Aged	t=0.3716	p=0.7188	Unpaired two-tailed t-test
Supp.Fig 1J	Young adults: WT vs hLRRK2*G2019S	t=0.2177	p=0.8316	Unpaired two-tailed t-test
	Aged: WT vs hLRRK2*G2019S	t=0.1268	p=0.9014	Unpaired two-tailed t-test
	WT: Young adults vs Aged	t=2.876	p=0.0139	Unpaired two-tailed t-test
	hLRRK2*G2019S: Young adults vs Aged	t=3.344	p=0.0074	Unpaired two-tailed t-test

Statistical report: Supplementary Figure 2.

Supplementary Figure 2				
**Figure**	**Experimental groups compared**	**Test statistic**	**P value**	**Statistical test**
Supp.Fig 2B	Saline: WT vs hLRRK2*G2019S	t=0.9347	p=0.3582	Unpaired two-tailed t-test
	MPTP 20 mg/kg 3 months: WT vs hLRRK2*G2019S	t=0.8524	p=0.4191	Unpaired two-tailed t-test with Welch's correction
	WT: Saline *vs* MPTP 20 mg/kg 3 months	t=0.8206	p=0.4214	Unpaired two-tailed t-test with Welch's correction
	hLRRK2*G2019S: Saline vs MPTP 20 mg/kg 3 months	t=0.9633	p=0.3475	Unpaired two-tailed t-test
Supp.Fig 2C	Saline: WT vs hLRRK2*G2019S	t=0.04350	p=0.9662	Unpaired two-tailed t-test
	MPTP 20 mg/kg 3 months: WT vs hLRRK2*G2019S	t=0.6367	p=0.5354	Unpaired two-tailed t-test
	WT: Saline *vs* MPTP 20 mg/kg 3 months	t=1.581	p=0.1399	Unpaired two-tailed t-test
	hLRRK2*G2019S: Saline vs MPTP 20 mg/kg 3 months	t=0.7105	p=0.4922	Unpaired two-tailed t-test
Supp.Fig 2D	Saline: WT vs hLRRK2*G2019S	t=0.2536	p=0.8018	Unpaired two-tailed t-test
	MPTP 20 mg/kg 3 months: WT vs hLRRK2*G2019S	t=0.2069	p=0.8415	Unpaired two-tailed t-test with Welch's correction
	WT: Saline *vs* MPTP 20 mg/kg 3 months	t=4.035	p=0.0007	Unpaired two-tailed t-test with Welch's correction
	hLRRK2*G2019S: Saline vs MPTP 20 mg/kg 3 months	t=2.672	p=0.0151	Unpaired two-tailed t-test
Supp.Fig 2E	Saline: WT vs hLRRK2*G2019S	t=0.2007	p=0.8450	Unpaired two-tailed t-test
	MPTP 20 mg/kg 3 months: WT vs hLRRK2*G2019S	t=0.5845	p=0.5689	Unpaired two-tailed t-test
	WT: Saline *vs* MPTP 20 mg/kg 3 months	t=1.853	p=0.0886	Unpaired two-tailed t-test
	hLRRK2*G2019S: Saline vs MPTP 20 mg/kg 3 months	t=1.272	p=0.2296	Unpaired two-tailed t-test
Supp.Fig 2F	Saline: WT vs hLRRK2*G2019S	t=1.141	p=0.2640	Unpaired two-tailed t-test
	MPTP 20 mg/kg 3 months: WT vs hLRRK2*G2019S	t=0.7680	p=0.4647	Unpaired two-tailed t-test with Welch's correction
	WT: Saline *vs* MPTP 20 mg/kg 3 months	t=0.7878	p=0.4398	Unpaired two-tailed t-test with Welch's correction
	hLRRK2*G2019S: Saline vs MPTP 20 mg/kg 3 months	t=1.060	p=0.3026	Unpaired two-tailed t-test
Supp.Fig 2G	Saline: WT vs hLRRK2*G2019S	t=0.05128	p=0.9601	Unpaired two-tailed t-test
	MPTP 20 mg/kg 3 months: WT vs hLRRK2*G2019S	U=20.50	p=0.4113	Mann–Whitney U test
	WT: Saline *vs* MPTP 20 mg/kg 3 months	t=1.573	p=0.1418	Unpaired two-tailed t-test
	hLRRK2*G2019S: Saline vs MPTP 20 mg/kg 3 months	t=0.7191	p=0.4871	Unpaired two-tailed t-test
Supp.Fig 2H	Saline: WT vs hLRRK2*G2019S	t=0.9209	p=0.3653	Unpaired two-tailed t-test
	MPTP 20 mg/kg 3 months: WT vs hLRRK2*G2019S	t=0.6305	p=0.5468	Unpaired two-tailed t-test with Welch's correction
	WT: Saline *vs* MPTP 20 mg/kg 3 months	t=0.7809	p=0.4436	Unpaired two-tailed t-test
	hLRRK2*G2019S: Saline vs MPTP 20 mg/kg 3 months	t=1.985	p=0.0618	Unpaired two-tailed t-test
Supp.Fig 2I	Saline: WT vs hLRRK2*G2019S	t=0.4784	p=0.6426	Unpaired two-tailed t-test
	MPTP 20 mg/kg 3 months: WT vs hLRRK2*G2019S	t=0.2435	p=0.8114	Unpaired two-tailed t-test
	WT: Saline *vs* MPTP 20 mg/kg 3 months	t=1.554	p=0.1462	Unpaired two-tailed t-test
	hLRRK2*G2019S: Saline vs MPTP 20 mg/kg 3 months	t=1.084	p=0.3014	Unpaired two-tailed t-test
Supp.Fig 2J	Saline: WT vs hLRRK2*G2019S	t=0.6526	p=0.5195	Unpaired two-tailed t-test
	MPTP 20 mg/kg 3 months: WT vs hLRRK2*G2019S	t=0.4514	p=0.6591	Unpaired two-tailed t-test
	WT: Saline *vs* MPTP 20 mg/kg 3 months	t=2.649	p=0.0150	Unpaired two-tailed t-test
	hLRRK2*G2019S: Saline vs MPTP 20 mg/kg 3 months	t=2.785	p=0.0118	Unpaired two-tailed t-test
Supp.Fig 2K	Saline: WT vs hLRRK2*G2019S	t=0.5261	p=0.6103	Unpaired two-tailed t-test
	MPTP 20 mg/kg 3 months: WT vs hLRRK2*G2019S	t=0.1565	p=0.8780	Unpaired two-tailed t-test
	WT: Saline *vs* MPTP 20 mg/kg 3 months	t=1.543	p=0.1489	Unpaired two-tailed t-test
	hLRRK2*G2019S: Saline vs MPTP 20 mg/kg 3 months	t=1.155	p=0.2726	Unpaired two-tailed t-test
Supp.Fig 2L	Saline: WT vs hLRRK2*G2019S	t=0.8266	p=0.4157	Unpaired two-tailed t-test
	MPTP 20 mg/kg 3 months: WT vs hLRRK2*G2019S	t=1.044	p=0.3156	Unpaired two-tailed t-test
	WT: Saline *vs* MPTP 20 mg/kg 3 months	t=3.226	p=0.0043	Unpaired two-tailed t-test with Welch's correction
	hLRRK2*G2019S: Saline vs MPTP 20 mg/kg 3 months	t=1.029	p=0.3162	Unpaired two-tailed t-test
Supp.Fig 2M	Saline: WT vs hLRRK2*G2019S	t=0.1952	p=0.8491	Unpaired two-tailed t-test
	MPTP 20 mg/kg 3 months: WT vs hLRRK2*G2019S	t=0.4236	p=0.6788	Unpaired two-tailed t-test
	WT: Saline *vs* MPTP 20 mg/kg 3 months	t=1.274	p=0.2268	Unpaired two-tailed t-test
	hLRRK2*G2019S: Saline vs MPTP 20 mg/kg 3 months	t=0.6474	p=0.5307	Unpaired two-tailed t-test
Supp.Fig 2N	Saline: WT vs hLRRK2*G2019S	t=1.066	p=0.2957	Unpaired two-tailed t-test
	MPTP 20 mg/kg 3 months: WT vs hLRRK2*G2019S	t=0.8383	p=0.4170	Unpaired two-tailed t-test
	WT: Saline *vs* MPTP 20 mg/kg 3 months	t=2.022	p=0.0561	Unpaired two-tailed t-test
	hLRRK2*G2019S: Saline vs MPTP 20 mg/kg 3 months	t=1.667	p=0.1119	Unpaired two-tailed t-test
Supp.Fig 2O	Saline: WT vs hLRRK2*G2019S	t=1.660	p=0.1279	Unpaired two-tailed t-test
	MPTP 20 mg/kg 3 months: WT vs hLRRK2*G2019S	t=0.1784	p=0.8612	Unpaired two-tailed t-test
	WT: Saline *vs* MPTP 20 mg/kg 3 months	t=0.1565	p=0.8782	Unpaired two-tailed t-test
	hLRRK2*G2019S: Saline vs MPTP 20 mg/kg 3 months	t=2.014	p=0.0691	Unpaired two-tailed t-test
Supp.Fig 2P	Saline: WT vs hLRRK2*G2019S	t=2.173	p=0.0387	Unpaired two-tailed t-test
	MPTP 20 mg/kg 3 months: WT vs hLRRK2*G2019S	t=0.2998	p=0.7690	Unpaired two-tailed t-test
	WT: Saline *vs* MPTP 20 mg/kg 3 months	t=1.194	p=0.2459	Unpaired two-tailed t-test
	hLRRK2*G2019S: Saline vs MPTP 20 mg/kg 3 months	t=3.180	p=0.0049	Unpaired two-tailed t-test
Supp.Fig 2Q	Saline: WT vs hLRRK2*G2019S	t=1.879	p=0.0896	Unpaired two-tailed t-test
	MPTP 20 mg/kg 3 months: WT vs hLRRK2*G2019S	t=1.509	p=0.1551	Unpaired two-tailed t-test
	WT: Saline *vs* MPTP 20 mg/kg 3 months	t=1.682	p=0.1183	Unpaired two-tailed t-test
	hLRRK2*G2019S: Saline vs MPTP 20 mg/kg 3 months	t=1.663	p=0.1244	Unpaired two-tailed t-test

Statistical report: Supplementary Figures 3-4.

Supplementary Figure 3					
**Figure**	**Experimental groups compared**	**Test statistic**	**P value**	**Post hoc P values**	**Statistical test**
Supp.Fig3A	Saline: WT vs hLRRK2*G2019S	t=2.011	p=0.0518		Unpaired two-tailed t-test
	MPTP 20mg/kg 1 month: WT vs hLRRK2*G2019S	t=0.09481	p=0.9255		Unpaired two-tailed t-test
	MPTP 20mg/kg 3 months: WT vs hLRRK2*G2019S	t=2.539	p=0.0227		Unpaired two-tailed t-test
	WT: Saline vs MPTP 20mg/kg (1 month, 3 months)	F(2,38)=1.900	p=0.1635	p=0.7423, p=0.2365	ANOVA test (Dunnett's post hoc test)
	hLRRK2*G2019S: Saline vs MPTP 20mg/kg (1 month, 3 months)	F(2,32)=3.402	p=0.0458	p=0.1573, p=0.0391	ANOVA test (Dunnett's post hoc test)
Supp.Fig3B	Saline: WT vs hLRRK2*G2019S	t=0.04220	p=0.9672		Unpaired two-tailed t-test
	MPTP 5mg/kg 1 month: WT vs hLRRK2*G2019S	t=1.034	p=0.3202		Unpaired two-tailed t-test
	WT: Saline vs MPTP 5mg/kg (1 month)	t=0.2597	p=0.7995		Unpaired two-tailed t-test
	hLRRK2*G2019S: Saline vs MPTP 5mg/kg (1 month)	t=0.4847	p=0.6374		Unpaired two-tailed t-test
Supp.Fig3C	Saline: WT vs hLRRK2*G2019S	t=2.074	p=0.0453		Unpaired two-tailed t-test
	MPTP 20mg/kg 1 month: WT vs hLRRK2*G2019S	U=40.50	p=0.3207		Mann-Whitney U test
	MPTP 20mg/kg 3 months: WT vs hLRRK2*G2019S	t=1.581	p=0.1348		Unpaired two-tailed t-test
	WT: Saline vs MPTP 20mg/kg (1 month, 3 months)	F(2,38)=7.771	p=0.0015	p=0.9970, p=0.0011	ANOVA test (Dunnett's post hoc test)
	hLRRK2*G2019S: Saline vs MPTP 20mg/kg (1 month, 3 months)	H=0.8253	p=0.6619	p>0.9999, p=0.7434	Kruskal-Wallis test (Dunn's post hoc test)
Supp.Fig3D	Saline: WT vs hLRRK2*G2019S	t=3.053	p=0.0122		Unpaired two-tailed t-test
	MPTP 5mg/kg 1 month: WT vs hLRRK2*G2019S	t=1.597	p=0.1344		Unpaired two-tailed t-test
	WT: Saline vs MPTP 5mg/kg (1 month)	t=0.2549	p=0.8031		Unpaired two-tailed t-test
	hLRRK2*G2019S: Saline vs MPTP 5mg/kg (1 month)	t=1.402	p=0.1884		Unpaired two-tailed t-test
Supp.Fig3E	Saline: WT vs hLRRK2*G2019S	t=2.261	p=0.0299		Unpaired two-tailed t-test
	MPTP 20mg/kg 1 month: WT vs hLRRK2*G2019S	t=0.1749	p=0.8630		Unpaired two-tailed t-test
	MPTP 20mg/kg 3 months: WT vs hLRRK2*G2019S	t=2.331	p=0.0341		Unpaired two-tailed t-test
	WT: Saline vs MPTP 20mg/kg (1 month, 3 months)	F(2,38)=1.380	p=0.2640	p=0.9703, p=0.2508	ANOVA test (Dunnett's post hoc test)
	hLRRK2*G2019S: Saline vs MPTP 20mg/kg (1 month, 3 months)	F(2,32)=3.157	p=0.0561	p=0.2014, p=0.0445	ANOVA test (Dunnett's post hoc test)
Supp.Fig3F	Saline: WT vs hLRRK2*G2019S	t=0.06608	p=0.9486		Unpaired two-tailed t-test
	MPTP 5mg/kg 1 month: WT vs hLRRK2*G2019S	t=1.219	p=0.2443		Unpaired two-tailed t-test
	WT: Saline vs MPTP 5mg/kg (1 month)	t=0.3758	p=0.7137		Unpaired two-tailed t-test
	hLRRK2*G2019S: Saline vs MPTP 5mg/kg (1 month)	t=0.3777	p=0.7128		Unpaired two-tailed t-test
Supp.Fig3G	Saline: WT vs hLRRK2*G2019S	t=2.861	p=0.0089		Unpaired two-tailed t-test with Welch's correction
	MPTP 20mg/kg 1 month: WT vs hLRRK2*G2019S	t=1.650	p=0.1154		Unpaired two-tailed t-test
	MPTP 20mg/kg 3 months: WT vs hLRRK2*G2019S	t=0.1684	p=0.8686		Unpaired two-tailed t-test
	WT: Saline vs MPTP 20mg/kg (1 month, 3 months)	W(2.000,12.71)=13.47	p=0.0007	p=0.0140, p=0.0039	Brown-Forsythe and Welch ANOVA test (Dunnett's T3 post hoc test)
	hLRRK2*G2019S: Saline vs MPTP 20mg/kg (1 month, 3 months)	F(2,32)=4.326	p=0.0217	p=0.0436, p=0.0353	ANOVA test (Dunnett's post hoc test)
Supp.Fig3H	Saline: WT vs hLRRK2*G2019S	t=1.160	p=0.2729		Unpaired two-tailed t-test
	MPTP 5mg/kg 1 month: WT vs hLRRK2*G2019S	t=0.8462	p=0.4128		Unpaired two-tailed t-test
	WT: Saline vs MPTP 5mg/kg (1 month)	t=0.1398	p=0.8911		Unpaired two-tailed t-test
	hLRRK2*G2019S: Saline vs MPTP 5mg/kg (1 month)	t=0.8404	p=0.4186		Unpaired two-tailed t-test

Supplementary Figure 4				
**Figure**	**Experimental groups compared**	**Test statistic**	**P value**	**Statistical test**
Supp.Fig4A	MPTP 20mg/kg 1 month: WT vs hLRRK2*G2019S	t=3.497	p=0.0044	Unpaired two-tailed t-test
	MPTP 20mg/kg 3 months: WT vs hLRRK2*G2019S	t=7.401	p=0.0001	Unpaired two-tailed t-test with Welch's correction
Supp.Fig4B	MPTP 5mg/kg 1 month: WT vs hLRRK2*G2019S	t=4.292	p=0.0027	Unpaired two-tailed t-test with Welch's correction
Supp.Fig4C	MPTP 20mg/kg 1 month: WT vs hLRRK2*G2019S	t=3.886	p=0.0025	Unpaired two-tailed t-test
	MPTP 20mg/kg 3 months: WT vs hLRRK2*G2019S	t=6.129	p<0.0001	Unpaired two-tailed t-test
Supp.Fig4D	MPTP 5mg/kg 1 month: WT vs hLRRK2*G2019S	t=7.991	p<0.0001	Unpaired two-tailed t-test with Welch's correction
Supp.Fig4E	MPTP 20mg/kg 1 month: WT vs hLRRK2*G2019S	t=1.910	p=0.0826	Unpaired two-tailed t-test
	MPTP 20mg/kg 3 months: WT vs hLRRK2*G2019S	t=4.699	p=0.0007	Unpaired two-tailed t-test
Supp.Fig4F	MPTP 5mg/kg 1 month: WT vs hLRRK2*G2019S	t=0.5440	p=0.5957	Unpaired two-tailed t-test
Supp.Fig4G	MPTP 20mg/kg 1 month: WT vs hLRRK2*G2019S	t=0.7366	p=0.4768	Unpaired two-tailed t-test
	MPTP 20mg/kg 3 months: WT vs hLRRK2*G2019S	t=2.168	p=0.0530	Unpaired two-tailed t-test
Supp.Fig4H	MPTP 5mg/kg 1 month: WT vs hLRRK2*G2019S	t=0.3456	p=0.7352	Unpaired two-tailed t-test

Statistical report: Supplementary Table 1.

Supplementary Table 1						
	Gene, MPTP treatment	Experimental groups compared	Test statistic	P value	Post hoc P values	Statistical test
Striatum	*Il6* , MPTP 20 mg/kg	MPTP 20mg/kg 1 month: WT vs hLRRK2*G2019S	t=1.560	p=0.1447		Unpaired two-tailed t-test
		MPTP 20mg/kg 3 months: WT vs hLRRK2*G2019S	U=19	p=0.8357		Mann-Whitney U test
		WT: Saline vs MPTP 20mg/kg (1 month, 3 months)	F(2,18)=1.793	p=0.1949	p=0.8414, p=0.1461	ANOVA test (Dunnett's post hoc test)
		hLRRK2*G2019S: Saline vs MPTP 20mg/kg (1 month, 3 months)	F(2,18)=2.873	p=0.0826	p=0.6302, p=0.2066	ANOVA test (Dunnett's post hoc test)
	Il6, MPTP 5 mg/kg	MPTP 5mg/kg 1 month: WT vs hLRRK2*G2019S	t=0.1506	p=0.8826		Unpaired two-tailed t-test
		WT: Saline vs MPTP 5mg/kg (1 month)	t=1.761	p=0.1036		Unpaired two-tailed t-test
		hLRRK2*G2019S: Saline vs MPTP 5mg/kg (1 month)	t=1.497	p=0.1625		Unpaired two-tailed t-test
	Ifng , MPTP 20 mg/kg	MPTP 20mg/kg 1 month: WT vs hLRRK2*G2019S	t=1.386	p=0.1910		Unpaired two-tailed t-test
		MPTP 20mg/kg 3 months: WT vs hLRRK2*G2019S	t=1.272	p=0.2322		Unpaired two-tailed t-test
		WT: Saline vs MPTP 20mg/kg (1 month, 3 months)	F(2,17)=1.894	p=0.1808	p=0.1243, p=0.7086	ANOVA test (Dunnett's post hoc test)
		hLRRK2*G2019S: Saline vs MPTP 20mg/kg (1 month, 3 months)	H=2.220	p=0.3415	p=0.4274, >0.9999	Kruskal-Wallis test (Dunn's post hoc test)
	Ifng, MPTP 5 mg/kg	MPTP 5mg/kg 1 month: WT vs hLRRK2*G2019S	t=0.8963	p=0.3877		Unpaired two-tailed t-test
		WT: Saline vs MPTP 5mg/kg (1 month)	t=1.252	p=0.2345		Unpaired two-tailed t-test
		hLRRK2*G2019S: Saline vs MPTP 5mg/kg (1 month)	t=0.5793	p=0.5752		Unpaired two-tailed t-test
	Tgfb1 , MPTP 20 mg/kg	MPTP 20mg/kg 1 month: WT vs hLRRK2*G2019S	t=0.3411	p=0.7390		Unpaired two-tailed t-test
		MPTP 20mg/kg 3 months: WT vs hLRRK2*G2019S	t=0.6585	p=0.5238		Unpaired two-tailed t-test
		WT: Saline vs MPTP 20mg/kg (1 month, 3 months)	F(2,18)=2.222	p=0.1373	p=0.7202, p=0.3025	ANOVA test (Dunnett's post hoc test)
		hLRRK2*G2019S: Saline vs MPTP 20mg/kg (1 month, 3 months)	F(2,18)=1.259	p=0.3078	p=0.8108, p=0.4697	ANOVA test (Dunnett's post hoc test)
	Tgfb1, MPTP 5 mg/kg	MPTP 5mg/kg 1 month: WT vs hLRRK2*G2019S	t=1.116	p=0.2846		Unpaired two-tailed t-test
		WT: Saline vs MPTP 5mg/kg (1 month)	t=1.452	p=0.1720		Unpaired two-tailed t-test
		hLRRK2*G2019S: Saline vs MPTP 5mg/kg (1 month)	t=1.043	p=0.3191		Unpaired two-tailed t-test
	Il4 , MPTP 20 mg/kg	MPTP 20mg/kg 1 month: WT vs hLRRK2*G2019S	U=24	p>0.9999		Mann-Whitney U test
		MPTP 20mg/kg 3 months: WT vs hLRRK2*G2019S	t=1.013	p=0.3351		Unpaired two-tailed t-test
		WT: Saline vs MPTP 20mg/kg (1 month, 3 months)	F(2,17)=0.9418	p=0.4093	p=0.6476, p=0.7764	ANOVA test (Dunnett's post hoc test)
		hLRRK2*G2019S: Saline vs MPTP 20mg/kg (1 month, 3 months)	H=0.04037	p=0.9823	p>0.9999, p>0.9999	Kruskal-Wallis test (Dunn's post hoc test)
	Il4, MPTP 5 mg/kg	MPTP 5mg/kg 1 month: WT vs hLRRK2*G2019S	U=23	p=0.6126		Mann-Whitney U test
		WT: Saline vs MPTP 5mg/kg (1 month)	t=0.1770	p=0.8624		Unpaired two-tailed t-test
		hLRRK2*G2019S: Saline vs MPTP 5mg/kg (1 month)	t=0.06253	p=0.9513		Unpaired two-tailed t-test
	Bdnf , MPTP 20 mg/kg	MPTP 20mg/kg 1 month: WT vs hLRRK2*G2019S	t=0.04947	p=0.9614		Unpaired two-tailed t-test
		MPTP 20mg/kg 3 months: WT vs hLRRK2*G2019S	t=1.247	p=0.2562		Unpaired two-tailed t-test with Welch's correction
		WT: Saline vs MPTP 20mg/kg (1 month, 3 months)	F(2,18)=2.738	p=0.0916	p=0.2629, p=0.6196	ANOVA test (Dunnett's post hoc test)
		hLRRK2*G2019S: Saline vs MPTP 20mg/kg (1 month, 3 months)	F(2,18)=3.129	p=0.0682	p=0.0677, p=0.9991	ANOVA test (Dunnett's post hoc test)
	Bdnf, MPTP 5 mg/kg	MPTP 5mg/kg 1 month: WT vs hLRRK2*G2019S	t=0.9976	p=0.3367		Unpaired two-tailed t-test
		WT: Saline vs MPTP 5mg/kg (1 month)	t=2.060	p=0.0617		Unpaired two-tailed t-test
		hLRRK2*G2019S: Saline vs MPTP 5mg/kg (1 month)	t=1.360	p=0.2012		Unpaired two-tailed t-test
Ventral mesencephalon	*Il6* , MPTP 20 mg/kg	MPTP 20mg/kg 1 month: WT vs hLRRK2*G2019S	t=1.263	p=0.2307		Unpaired two-tailed t-test
		MPTP 20mg/kg 3 months: WT vs hLRRK2*G2019S	t=1.961	p=0.0735		Unpaired two-tailed t-test
		WT: Saline vs MPTP 20mg/kg (1 month, 3 months)	F(2,19)=0.9166	p=0.4168	p=0.7876, p=0.6759	ANOVA test (Dunnett's post hoc test)
		hLRRK2*G2019S: Saline vs MPTP 20mg/kg (1 month, 3 months)	F(2,18)=7.714	p=0.0038	p=0.1711, p=0.0545	ANOVA test (Dunnett's post hoc test)
	Il6, MPTP 5 mg/kg	MPTP 5mg/kg 1 month: WT vs hLRRK2*G2019S	t=2.162	p=0.0516		Unpaired two-tailed t-test
		WT: Saline vs MPTP 5mg/kg (1 month)	t=1.349	p=0.2046		Unpaired two-tailed t-test
		hLRRK2*G2019S: Saline vs MPTP 5mg/kg (1 month)	t=0.4127	p=0.6885		Unpaired two-tailed t-test
	Ifng , MPTP 20 mg/kg	MPTP 20mg/kg 1 month: WT vs hLRRK2*G2019S	t=0.3678	p=0.7195		Unpaired two-tailed t-test
		MPTP 20mg/kg 3 months: WT vs hLRRK2*G2019S	U=18	p=0.4557		Mann-Whitney U test
		WT: Saline vs MPTP 20mg/kg (1 month, 3 months)	F(2,20)=0.3674	p=0.6971	p=0.9885, p=0.7250	ANOVA test (Dunnett's post hoc test)
		hLRRK2*G2019S: Saline vs MPTP 20mg/kg (1 month, 3 months)	F(2,18)=0.06658	p=0.9358	p=0.9343, p=0.9993	ANOVA test (Dunnett's post hoc test)
	Ifng, MPTP 5 mg/kg	MPTP 5mg/kg 1 month: WT vs hLRRK2*G2019S	t=0.09282	p=0.9275		Unpaired two-tailed t-test
		WT: Saline vs MPTP 5mg/kg (1 month)	t=0.3339	p=0.7442		Unpaired two-tailed t-test
		hLRRK2*G2019S: Saline vs MPTP 5mg/kg (1 month)	t=0.4374	p=0.6703		Unpaired two-tailed t-test
	Tgfb1 , MPTP 20 mg/kg	MPTP 20mg/kg 1 month: WT vs hLRRK2*G2019S	t=0.1383	p=0.8923		Unpaired two-tailed t-test
		MPTP 20mg/kg 3 months: WT vs hLRRK2*G2019S	t=0.5954	p=0.5648		Unpaired two-tailed t-test
		WT: Saline vs MPTP 20mg/kg (1 month, 3 months)	F(2,18)=1.997	p=0.1647	p=0.1138, p=0.4523	ANOVA test (Dunnett's post hoc test)
		hLRRK2*G2019S: Saline vs MPTP 20mg/kg (1 month, 3 months)	F(2,18)=0.8884	p=0.4286	p=0.3529, p=0.9406	ANOVA test (Dunnett's post hoc test)
	Tgfb1, MPTP 5 mg/kg	MPTP 5mg/kg 1 month: WT vs hLRRK2*G2019S	t=0.6512	p=0.5263		Unpaired two-tailed t-test
		WT: Saline vs MPTP 5mg/kg (1 month)	t=0.9179	p=0.3768		Unpaired two-tailed t-test
		hLRRK2*G2019S: Saline vs MPTP 5mg/kg (1 month)	t=0.7327	p=0.4791		Unpaired two-tailed t-test
	Il4 , MPTP 20 mg/kg	MPTP 20mg/kg 1 month: WT vs hLRRK2*G2019S	U=24	p=0.6943		Mann-Whitney U test
		MPTP 20mg/kg 3 months: WT vs hLRRK2*G2019S	t=0.8594	p=0.4070		Unpaired two-tailed t-test
		WT: Saline vs MPTP 20mg/kg (1 month, 3 months)	F(2,20)=0.5247	p=0.5997	p=0.5419, p=0.9841	ANOVA test (Dunnett's post hoc test)
		hLRRK2*G2019S: Saline vs MPTP 20mg/kg (1 month, 3 months)	H=0.7093	p=0.7154	p>0.9999, p=0.8204	Kruskal-Wallis test (Dunn's post hoc test)
	Il4, MPTP 5 mg/kg	MPTP 5mg/kg 1 month: WT vs hLRRK2*G2019S	t=2.224	p=0.0445		Unpaired two-tailed t-test
		WT: Saline vs MPTP 5mg/kg (1 month)	t=2.374	p=0.0536		Unpaired two-tailed t-test with Welch's correction
		hLRRK2*G2019S: Saline vs MPTP 5mg/kg (1 month)	t=1.522	p=0.1561		Unpaired two-tailed t-test
	Bdnf , MPTP 20 mg/kg	MPTP 20mg/kg 1 month: WT vs hLRRK2*G2019S	t=0.8955	p=0.3881		Unpaired two-tailed t-test
		MPTP 20mg/kg 3 months: WT vs hLRRK2*G2019S	t=1.675	p=0.1248		Unpaired two-tailed t-test
		WT: Saline vs MPTP 20mg/kg (1 month, 3 months)	F(2,18)=3.627	p=0.0475	p=0.0345, p=0.1572	ANOVA test (Dunnett's post hoc test)
		hLRRK2*G2019S: Saline vs MPTP 20mg/kg (1 month, 3 months)	F(2,18)=1.152	p=0.3381	p=0.3573, p=0.9848	ANOVA test (Dunnett's post hoc test)
	Bdnf, MPTP 5 mg/kg	MPTP 5mg/kg 1 month: WT vs hLRRK2*G2019S	t=0.3132	p=0.7591		Unpaired two-tailed t-test
		WT: Saline vs MPTP 5mg/kg (1 month)	t=0.5081	p=0.6206		Unpaired two-tailed t-test
		hLRRK2*G2019S: Saline vs MPTP 5mg/kg (1 month)	t=0.1795	p=0.8608		Unpaired two-tailed t-test
